# Supplementary material for: DHHC9-mediated GLUT1 S-palmitoylation promotes glioblastoma glycolysis and tumorigenesis
Source: Nat Commun. 2021 Oct 7;12:5872. doi: 10.1038/s41467-021-26180-4 (PMC8497546; doi:10.1038/s41467-021-26180-4)
Supplement: Supplementary file 1 — Supplementary Information [file 41467_2021_26180_MOESM1_ESM.pdf]

Supplementary Information

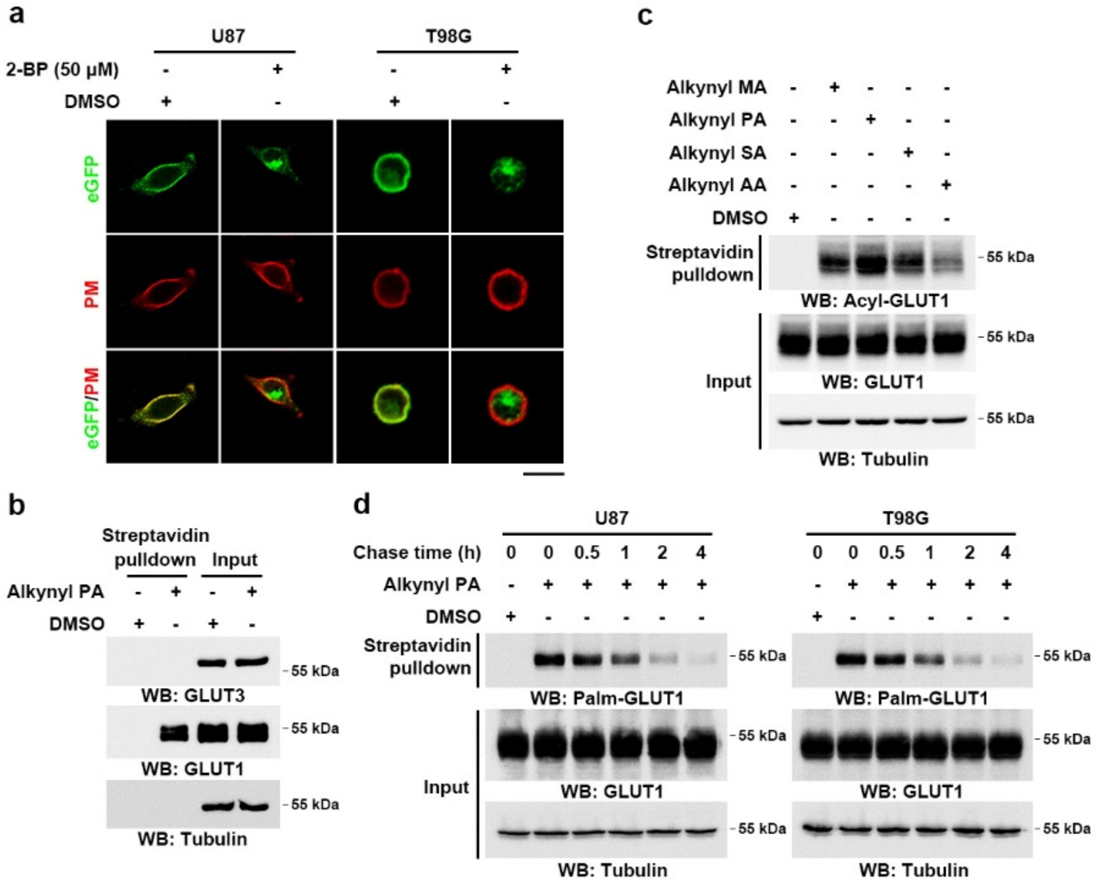

Supplementary Fig.1: PM localization of GLUT1 is regulated by S-palmitoylation.

(**a**) U87 or T98G cells transfected with plasmids expressing eGFP-GLUT1 were treated with dimethyl sulfoxide (DMSO) or 50 μM of 2-BP for 8 hours followed by incubation with 1 μg ml^{-1} of CT-B conjugated with Alexa Fluor 594 for 5 min at 37°C. The cells were then analyzed by fluorescence microscopy. PM, plasma membrane. Scale bar, 20 μm. (**b**) U87 cells were metabolically labelled with 50 μM of alkynyl PA for 4 hours. GLUT1 and GLUT3 palmitoylation levels were analyzed by click reaction

and streptavidin bead pulldown, followed by immunoblotting. (**c**) U87 cells were metabolically labelled with 50 μM of different chemical probes (alkynyl myristic acid [MA], alkynyl palmitic acid [PA], alkynyl stearic acid [SA], and alkynyl arachidonic acid [AA]) for 4 hours. GLUT1 fatty acylation levels (acylated GLUT1) were analyzed by click reaction and streptavidin bead pulldown, followed by immunoblotting. (**d**) U87 or T98G cells were metabolically labelled with 50 μM of alkynyl PA for 4 hours, and subsequently chased by the addition of an excess of 200 μM BSA-conjugated palmitic acid. Cells were collected for analyses of GLUT1 palmitoylation at the indicated time point. Representative results were obtained from at least three independent experiments with similar results. Source data are provided as a Source Data file.

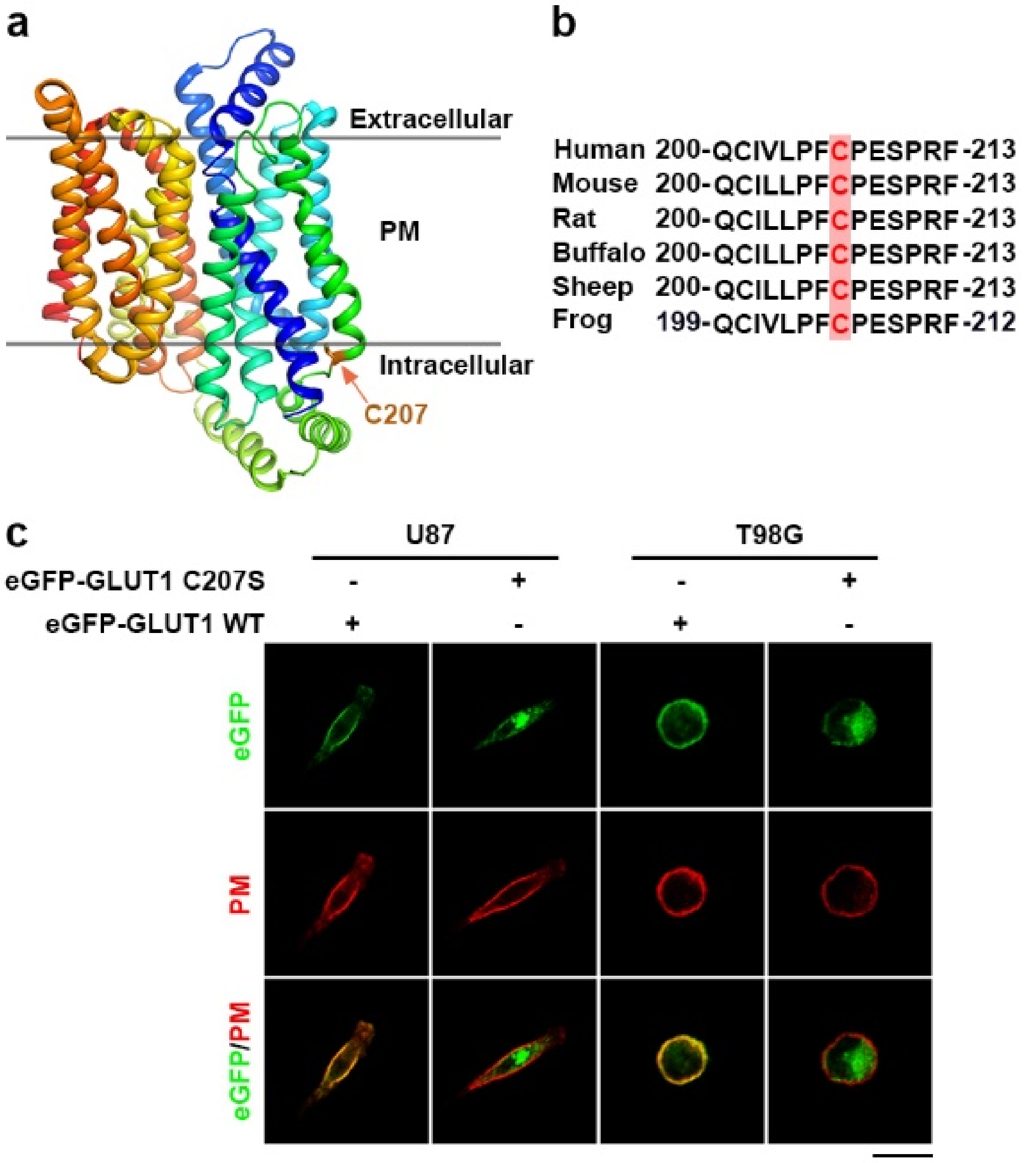

Supplementary Fig.2: Cys207 palmitoylation is evolutionarily conserved and is required for maintaining GLUT1 PM localization.

(**a**) Intramolecular location of putative palmitoylated amino acid residue was displayed in the 3-dimensional structural model of human GLUT1 (PDB code: 4PYP). Cys207 shown in orange is close to the inner PM. PM, plasma membrane. (**b**) Alignment of GLUT1 protein sequence among different species. (**c**) U87 or T98G cells transfected with plasmids

expressing WT eGFP-GLUT1 or eGFP-GLUT1 C207S were incubated with 1 μg ml^{-1} of CT-B conjugated with Alexa Fluor 594 for 5 min at 37 °C, the cells were then analyzed by fluorescence microscopy. PM, plasma membrane. Scale bar, 20 μm. Representative results were obtained from at least three independent experiments with similar results.

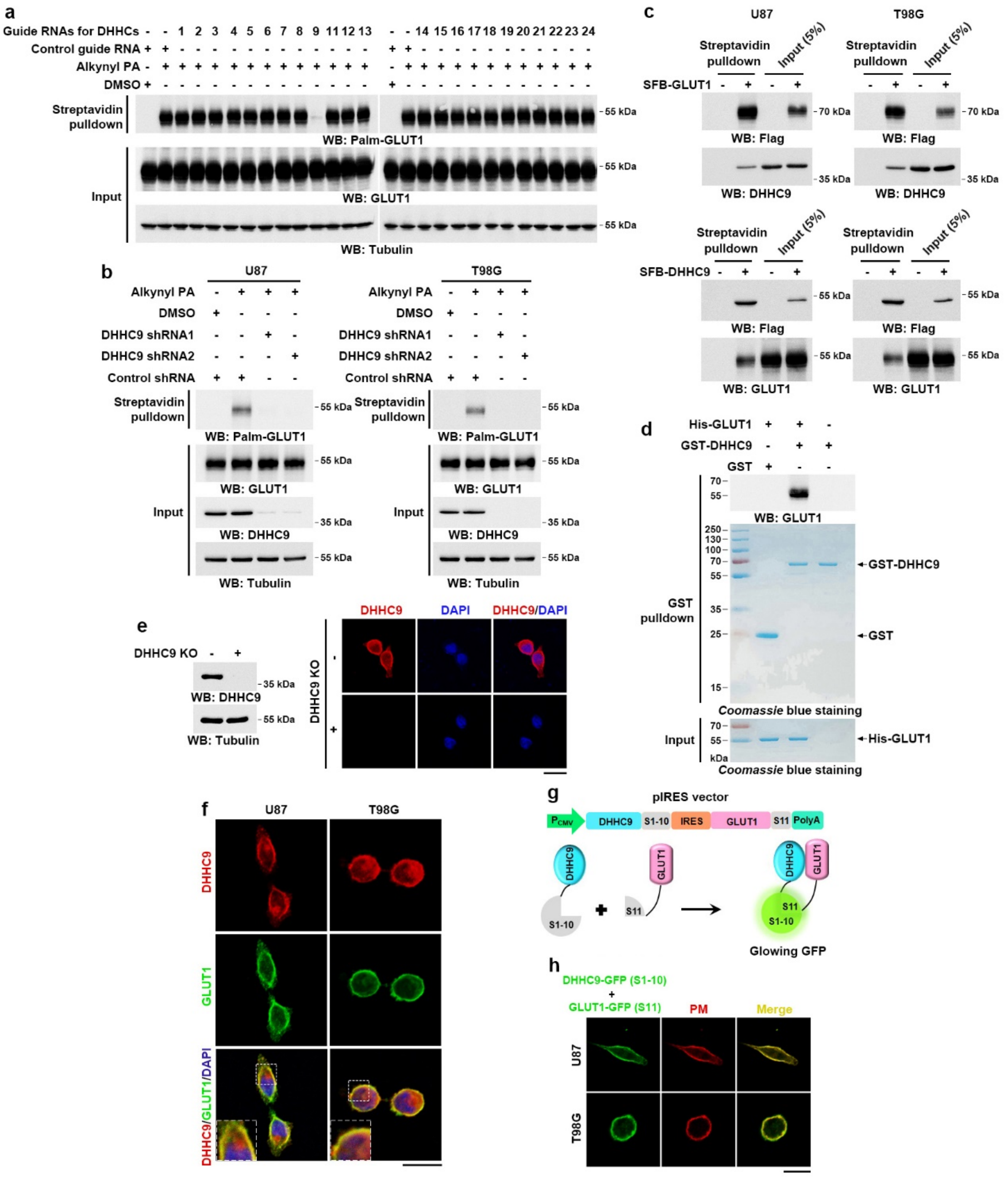

Supplementary Fig.3: DHHC9 is the palmitoyl acyltransferase of GLUT1.

(**a**) U87 cells infected with lentiviruses containing expression cassettes of control guide RNA or guide RNAs targeting different *DHHC*s and hSpCas9 protein were cultured with 1 μg ml^{-1} of puromycin for 7 days. The cells were metabolically labelled with 50 μM of alkynyl PA for 4 hours. GLUT1 palmitoylation levels were analyzed by click reaction and streptavidin bead pulldown, followed by immunoblotting. (**b**) U87 or T98G cells infected with lentiviruses expressing control shRNA or *DHHC9* shRNAs were metabolically labelled with 50 μM of alkynyl PA for 4 hours. GLUT1 palmitoylation levels were analyzed by click reaction and streptavidin bead pulldown, followed by immunoblotting. (**c**) U87 or T98G cells were transfected with empty vector or plasmids expressing S protein-Flag-Streptavidin binding peptide (SFB)-tagged GLUT1 or DHHC9. Streptavidin pulldown was performed 48 hours after transfection followed by immunoblotting using the indicated antibodies. (**d**) GST pulldown analysis was performed by mixing purified immobilized GST-DHHC9 on glutathione agarose beads with purified His-GLUT1. (**e**) U87 cells with or without knockout of endogenous DHHC9 were analyzed by immunoblotting (left panel) and immunofluorescent staining (right panel) using antibody against DHHC9. (**f**) Cellular localization of endogenous DHHC9 and GLUT1 was visualized by immunofluorescent staining using

anti-DHHC9 and anti-GLUT1 antibody, respectively. Square areas inside the white dash lines were enlarged and the yellow color indicates the colocalization of DHHC9 and GLUT1. (**g**) Diagram of the split-GFP system for determination of the colocalization of DHHC9 and GLUT1. A bicistronic cassette coexpressing C-terminal split-GFP (S1-10)-tagged DHHC9 and split-GFP (S11)-tagged GLUT1 was constructed (upper panel). Fluorescence occurs when split-GFP (S1-10)-tagged DHHC9 colocalizes with split-GFP (S11)-tagged GLUT1 (lower panel). (**h**) U87 or T98G cells transfected with plasmids coexpressing C-terminal split-GFP (S1-10)-tagged DHHC9 and split-GFP (S11)-tagged GLUT1 were incubated with 1 μg ml^{-1} of CT-B conjugated with Alexa Fluor 594 for 5 min at 37 °C 24 hours after transfection, the cells were then analyzed by fluorescence microscopy (lower panel). Scale bar, 20 μm (**e**, **f**, **h**). Representative results were obtained from at least three independent experiments with similar results. Source data are provided as a Source Data file.

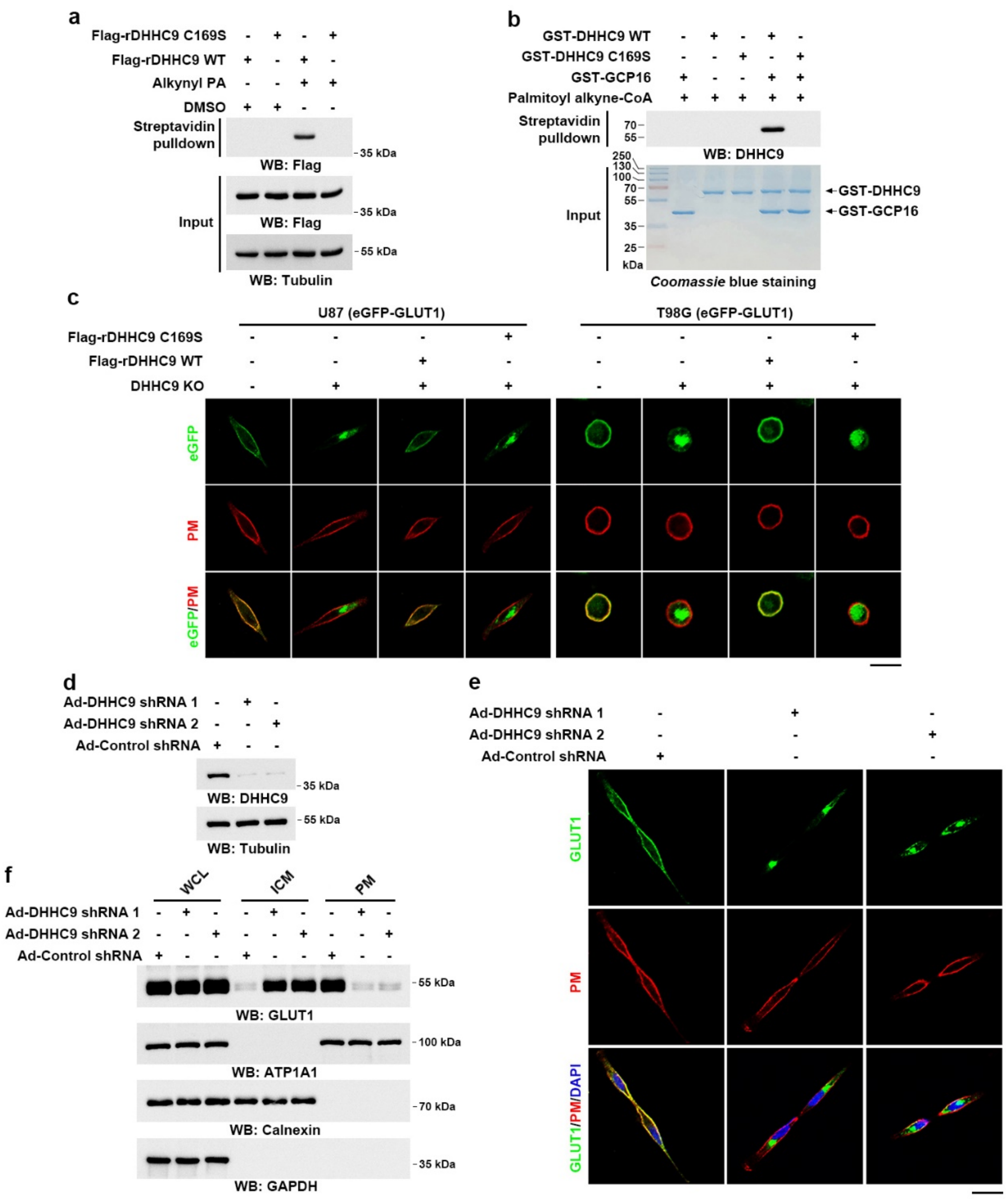

Supplementary Fig.4: DHHC9 is required for palmitoylation and PM localization of GLUT1 in GBM cells.

(**a**) U87 cells were transfected with Flag-tagged WT DHHC9 or the inactive DHHC9 C169S mutant. Twenty-four hours after transfection, the cells were metabolically labelled with 50 μM of alkynyl PA for 4 hours. Palmitoylation levels of Flag-DHHC9 were analyzed by click reaction and

streptavidin bead pulldown, followed by immunoblotting. (**b**) *In vitro* palmitoylation analysis was performed by incubating purified GST-DHHC9 and/or GST-GCP16 proteins with palmitoyl alkyne-CoA. DHHC9 autopalmitoylation was detected by click reaction and streptavidin bead pulldown, followed by immunoblotting. Indicated GST-DHHC9 and GST-GCP16 proteins were visualized by *Coomassie* blue staining. (**c**) DHHC9-knockout U87 or T98G cells with or without reconstituted expression of WT DHHC9 or DHHC9 C169S were transfected with plasmids expressing eGFP-GLUT1. Twenty-four hours after transfection, the cells were incubated with 1 µg ml^{-1} of CT-B conjugated with Alexa Fluor 594 for 5 min at 37 °C, and analyzed by fluorescence microscopy. (**d**) PDX cells infected with adenoviruses expressing control shRNA or *DHHC9* shRNAs were analyzed by immunoblotting using indicated antibodies 24 hours after infection. (**e**) PDX cells were infected with adenoviruses expressing control shRNA or *DHHC9* shRNAs. Twenty-four hours after infection, the cells were incubated with 1 µg ml^{-1} of CT- B conjugated with Alexa Fluor 594 for 5 min at 37 °C. GLUT1 cellular localization was visualized by immunofluorescent staining using antibody against GLUT1 and the plasma membrane was marked by Alexa Fluor 594-conjugated CT-B. PM, plasma membrane. (**f**) PDX cells were infected with adenoviruses expressing control shRNA or *DHHC9* shRNAs. Twenty-four hours after infection, levels of GLUT1 in PM and ICM fractions were analyzed by

immunoblotting. ATP1A1, Calnexin and GAPDH served as the marker of PM, ICM and cytosol fraction, respectively. PM, plasma membrane; ICM, intracellular membrane; WCL, whole cell lysate. Scale bar, 20 μm (**c**, **e**). Representative results were obtained from at least three independent experiments with similar results. Source data are provided as a Source Data file.

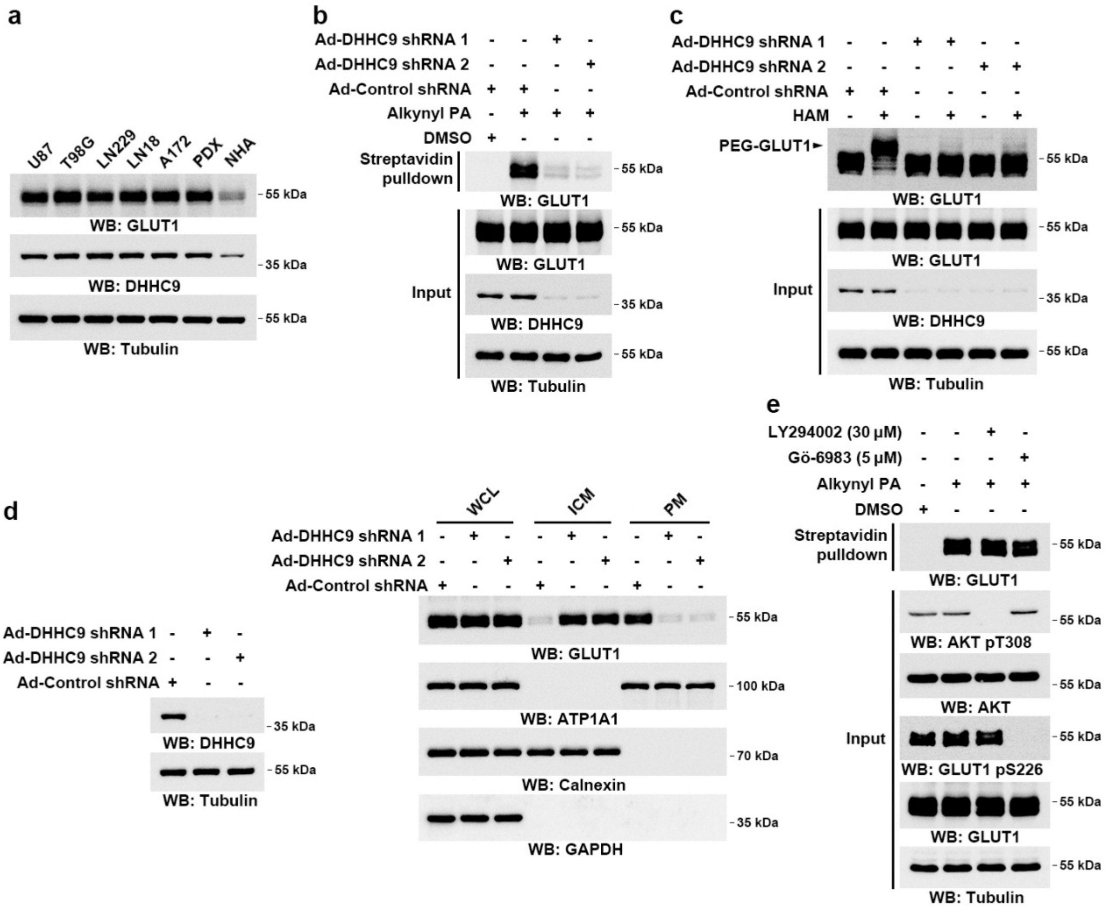

Supplementary Fig.5: DHHC9 is required for palmitoylation and PM localization of GLUT1 in normal astrocytes.

(**a**) Whole cell lysates prepared from indicated cells were analyzed with immunoblotting using the indicated antibodies. (**b**) NHAs were infected

with adenoviruses expressing control shRNA or *DHHC9* shRNAs. Twenty-four hours after infection, the cells were metabolically labelled with 50 μM of alkynyl PA for 4 hours. GLUT1 palmitoylation levels were analyzed by click reaction and streptavidin bead pulldown, followed by immunoblotting. (**c**) NHAs were infected with adenoviruses expressing control shRNA or *DHHC9* shRNAs. APE assay was performed to analyzed the GLUT1 palmitoylation 24 hours after infection. The top band indicates the palmitoylated GLUT1 (PEG-GLUT1). (**d**) NHAs were infected with adenoviruses expressing control shRNA or *DHHC9* shRNAs. Twenty-four hours after infection, endogenous DHHC9 levels in WCLs (left panel) and GLUT1 levels in PM and ICM fractions (right panel) were determined by immunoblotting using indicated antibodies. ATP1A1, Calnexin and GAPDH served as the marker of the PM, ICM, and cytosol fraction, respectively. Tubulin served as a loading control for WCL. PM, plasma membrane; ICM, intracellular membrane; WCL, whole cell lysate. (**e**) U87 cells pretreated with PI3K inhibitor LY294002 (30 μM) or PKC inhibitor Gö 6983 (5 μM) for 1 hour were metabolically labelled with 50 μM of alkynyl PA for 4 hours. GLUT1 palmitoylation levels were analyzed by click reaction and streptavidin bead pulldown, followed by immunoblotting. Representative results were obtained from at least three independent experiments with similar results. Source data are provided as a Source Data file.

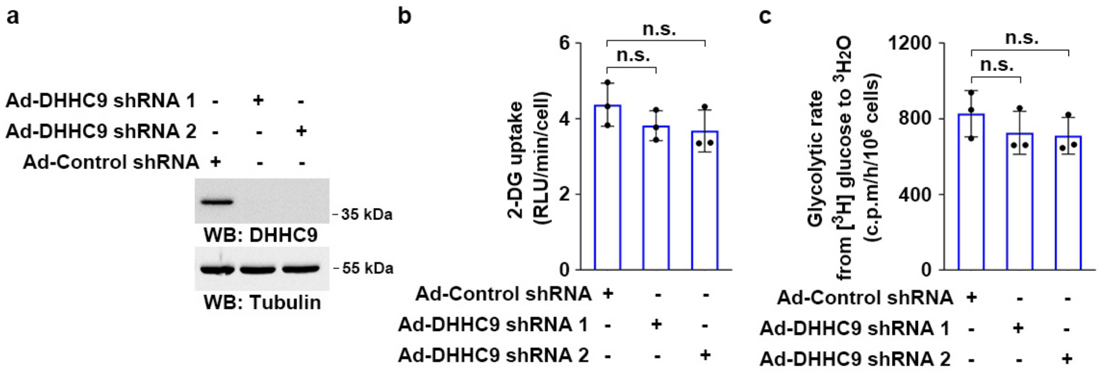

Supplementary Fig.6: DHHC9 depletion does not significantly alter glycolysis in normal astrocytes.

(**a-c**) NHAs were infected with adenoviruses expressing control shRNA or *DHHC9* shRNAs for 24 hours. Data represent the mean ± SD of three independent experiments. The two-tailed Student's *t*-test was performed to analyzed the differences between the control and experimental groups. n.s., not significant. (**a**) Endogenous DHHC9 levels in WCLs were determined by immunoblotting using indicated antibodies. WCLs, whole cell lysates. Representative results were obtained from at least three independent experiments with similar results. (**b**) The indicated cells were treated with 1 mM of 2-DG for 10 min. Uptake of 2-DG was measured using a glucose uptake kit and normalized to cell number. RLU, relative luminescence units. (**c**) The indicated cells were incubated with 5.5 mM glucose spiked with 10 μCi of D-[5-^{3}H] glucose for 1 hour. The glycolytic rate was measured by monitoring the conversion of D-[5-^{3}H] glucose to ^3H_2O and normalized to cell numbers. c.p.m., counts per minute. Source data are provided as a Source Data file.

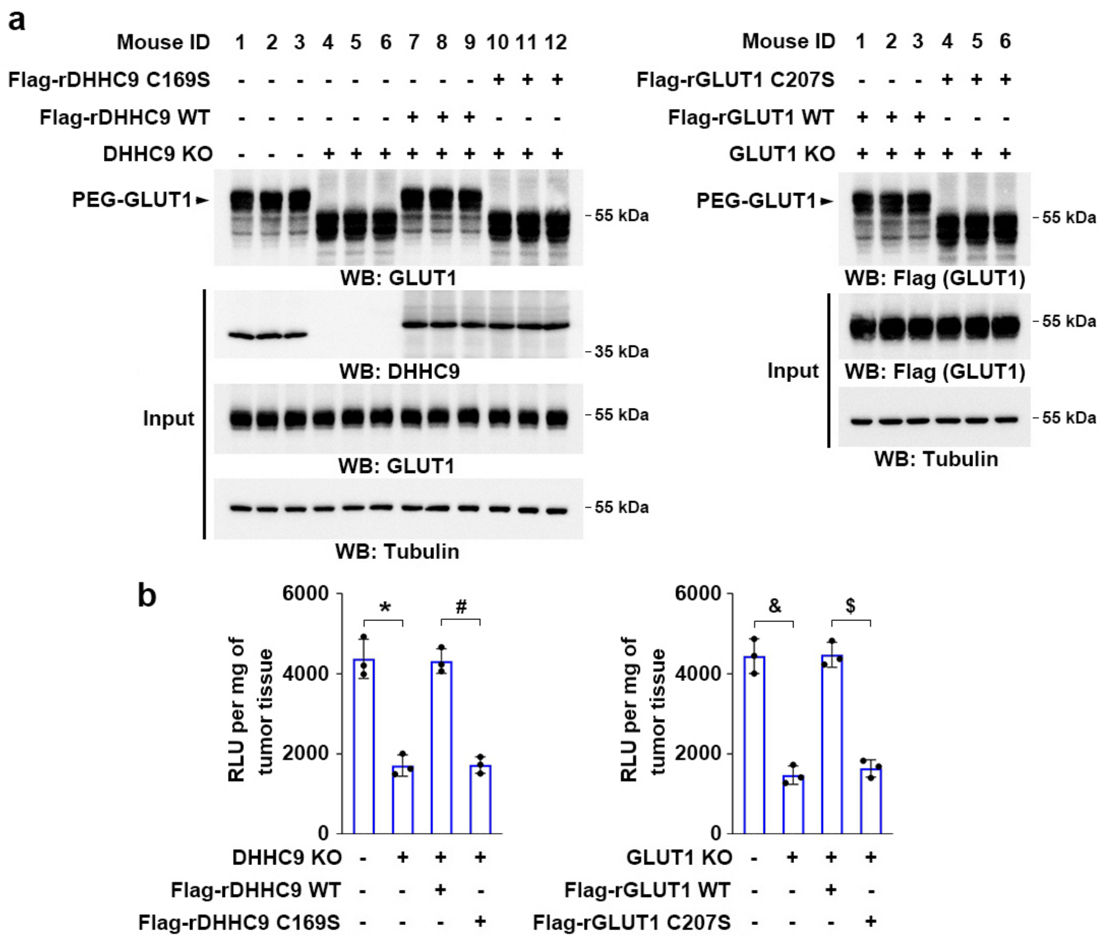

Supplementary Fig.7: DHHC9-mediated S-palmitoylation of GLUT1 promotes glucose uptake in tumor tissues.

(**a**) GLUT1 S-palmitoylation was analyzed by the APE assays in xenograft-tumors derived from U87 cells. The top band indicates the palmitoylated GLUT1 (PEG-GLUT1). (**b**) 2-DG (0.2 ml [500 mg/kg]) was intraperitoneally injected into brain tumor-bearing mice 6 hours before sacrifice. The tumor tissues (20 mg/each) from each group of mice (n = 3) were dissected for homogenization. Glucose uptake was measured and normalized to the tissue weight. Data were collected from n = 3 mice per group. The mean ± SD values are shown. *P* values were determined by

two-tailed Student's *t*-test. * *P* = 1.16E-03, # *P* = 2.66E-04, & *P* = 4.68E-04, $ *P* = 2.04E-04. RLU, relative luminescence units. Source data are provided as a Source Data file.

Supplementary Table 1. Multivariate analysis of overall survival in GBM patients (Cox's regression model).

Protein	Characteristics	HR	95% CI	*P* value
DHHC9	Age, years (>49 vs ≤49)	2.438	0.971-6.123	0.058
	Sex (female vs male)	1.522	0.716-3.236	0.275
	Total resection (yes vs no)	0.513	0.234-1.126	0.096
	DHHC9 staining scores (5–8 vs 0–4)	2.971	1.294-6.822	**0.01**
PM GLUT1	Age, years (>49 vs ≤49)	2.383	0.94-6.042	0.067
	Sex (female vs male)	1.264	0.612-2.612	0.527
	Total resection (yes vs no)	0.372	0.165-0.835	**0.017**
	PM GLUT1 staining scores (5–8 vs 0–4)	2.988	1.386-6.443	**0.005**

Abbreviation: HR, hazard ratio; CI, confidence interval; PM, plasma membrane. Bold *P* values are statistically significant.

Supplementary Table 2. The gRNA and shRNA information

Control gRNA	CGCTTCCGCGGCCCGTTCAA
DHHC1 gRNA	TGCGGGACAAGAGCTATGCG
DHHC2 gRNA	GTAGGACCAGCCGAGCAGGA
DHHC3 gRNA	ACCACTTCCGAAACATTGAG
DHHC4 gRNA	AGACCGTGTCCATAACATGG
DHHC5 gRNA	GAACCACGTGAAATCCCGTG
DHHC6 gRNA	GCAGCATGGATACAACCCAG
DHHC7 gRNA	CGTCACGGATGAACCAGACC
DHHC8 gRNA	TGAAAGGGTTCACACCCCCG
DHHC9 gRNA	TTGTCTGGTTGAGAGCCACG
DHHC11 gRNA	ACTCGTGTACACCCAGACGG
DHHC12 gRNA	GCTGGCGGAGATAGGCGATG
DHHC13 gRNA	GCTGTTGTAGATCAGTTGGG
DHHC14 gRNA	GTTCTACGCAGTTATCACAA
DHHC15 gRNA	AGTAGGACCAGAGCACGACG
DHHC16 gRNA	GCCAGCGCTGTACTAGACCC
DHHC17 gRNA	GTTTGATACGAAAACCGGTG
DHHC18 gRNA	GTACCAGCAGATCAGCCCCG
DHHC19 gRNA	AAGCAGGGAAGGGACCCCCG
DHHC20 gRNA	GTAGGACCAGACGACCACGA
DHHC21 gRNA	ACTGTTGTGAAGATATATCG
DHHC22 gRNA	GGAATAGGAAGAGCGCCCCG
DHHC23 gRNA	CAGTGTGATCCCATACACCG
DHHC24 gRNA	CACGCACGTGTCCGTCACGA
Control shRNA	GCTTCTAACACCGGAGGTCTT
DHHC9 shRNA1	ATCTGGAAATTCTTGATAC
DHHC9 shRNA2	TTCTTGATACGAGGCGGTG
