# Supplementary material for: DHHC9-mediated GLUT1 S-palmitoylation promotes glioblastoma glycolysis and tumorigenesis
Source: Nat Commun. 2021 Oct 7;12:5872. doi: 10.1038/s41467-021-26180-4 (PMC8497546; doi:10.1038/s41467-021-26180-4)
Supplement: Supplementary file 3 — Reporting summary [file 41467_2021_26180_MOESM3_ESM.pdf]

nature portfolio

Corresponding author(s): Xinjian Li

Last updated by author(s): Sep 2, 2021

Reporting Summary

Nature Portfolio wishes to improve the reproducibility of the work that we publish. This form provides structure for consistency and transparency in reporting. For further information on Nature Portfolio policies, see our Editorial Policies and the Editorial Policy Checklist.

Statistics

For all statistical analyses, confirm that the following items are present in the figure legend, table legend, main text, or Methods section.

n/a	Confirmed	
☐	☒	The exact sample size (*n*) for each experimental group/condition, given as a discrete number and unit of measurement
☐	☒	A statement on whether measurements were taken from distinct samples or whether the same sample was measured repeatedly
☐	☒	The statistical test(s) used AND whether they are one- or two-sided<br>*Only common tests should be described solely by name; describe more complex techniques in the Methods section.*
☒	☐	A description of all covariates tested
☐	☒	A description of any assumptions or corrections, such as tests of normality and adjustment for multiple comparisons
☐	☒	A full description of the statistical parameters including central tendency (e.g. means) or other basic estimates (e.g. regression coefficient) AND variation (e.g. standard deviation) or associated estimates of uncertainty (e.g. confidence intervals)
☐	☒	For null hypothesis testing, the test statistic (e.g. *F*, *t*, *r*) with confidence intervals, effect sizes, degrees of freedom and *P* value noted<br>*Give P values as exact values whenever suitable.*
☒	☐	For Bayesian analysis, information on the choice of priors and Markov chain Monte Carlo settings
☒	☐	For hierarchical and complex designs, identification of the appropriate level for tests and full reporting of outcomes
☐	☒	Estimates of effect sizes (e.g. Cohen's *d*, Pearson's *r*), indicating how they were calculated

Our web collection on statistics for biologists contains articles on many of the points above.

Software and code

Policy information about availability of computer code

Data collection	Bioluminescent imaging of mice was recorded using an IVIS Lumina System coupled with the Living Image data-acquisition software program version 4.7.3.
Data analysis	The intensity of immunoblotting bands was quantified by the Image Lab software program version 6.1. Photoshops version: 19.0 was used for quantification of immunofluorescence intensity. IBM SPSS Statistics version: 23.0 was used to perform the two-tailed Pearson correlation analysis, the multivariate analysis, the two-tailed log-rank tests, and the two-tailed Student t-tests. The peak photon flux within a region of mouse was recorded and quantified using an IVIS Lumina System coupled with the Living Image data-acquisition software program version 4.7.3.

For manuscripts utilizing custom algorithms or software that are central to the research but not yet described in published literature, software must be made available to editors and reviewers. We strongly encourage code deposition in a community repository (e.g. GitHub). See the Nature Portfolio guidelines for submitting code & software for further information.

Data

Policy information about availability of data

All manuscripts must include a data availability statement. This statement should provide the following information, where applicable:

- Accession codes, unique identifiers, or web links for publicly available datasets
- A description of any restrictions on data availability
- For clinical datasets or third party data, please ensure that the statement adheres to our policy

The authors declare that [the/all other] data supporting the findings of this study are available within the paper and its supplementary information files. Source data are provided with this paper.

Field-specific reporting

Please select the one below that is the best fit for your research. If you are not sure, read the appropriate sections before making your selection.

- [x] Life sciences
- [ ] Behavioural & social sciences
- [ ] Ecological, evolutionary & environmental sciences

For a reference copy of the document with all sections, see nature.com/documents/nr-reporting-summary-flat.pdf

Life sciences study design

All studies must disclose on these points even when the disclosure is negative.

Sample size	We used online tools available at http://www.biomath.info/power/ttest.htm to determine the sample or group sizes of the experiments.
Data exclusions	No data were excluded from analysis.
Replication	Results were confirmed in at least three biological replicates for each experiment unless otherwise stated.
Randomization	The samples for each experiment were randomized to be examined (No specific methods were used).
Blinding	No blinding was performed due to none of the analyses reported involved procedures that could be influenced by investigator bias.

Reporting for specific materials, systems and methods

We require information from authors about some types of materials, experimental systems and methods used in many studies. Here, indicate whether each material, system or method listed is relevant to your study. If you are not sure if a list item applies to your research, read the appropriate section before selecting a response.

Materials & experimental systems

n/a	Involved in the study	
☐	☒	Antibodies
☐	☒	Eukaryotic cell lines
☒	☐	Palaeontology and archaeology
☐	☒	Animals and other organisms
☐	☒	Human research participants
☒	☐	Clinical data
☒	☐	Dual use research of concern

Methods

n/a	Involved in the study	
☒	☐	ChIP-seq
☒	☐	Flow cytometry
☒	☐	MRI-based neuroimaging

Antibodies

Antibodies used

Rabbit monoclonal antibodies recognizing GLUT1 (clone name: EPR3915, #ab115730, lot: GR 3266142-4) for immunoblotting/immunofluorescent staining was purchased from Abcam
Rabbit monoclonal antibodies recognizing tubulin (clone name: EPR13478(B), #ab176560, lot: GR 177622-63) for immunoblotting was purchased from Abcam
Rabbit monoclonal antibodies recognizing Ki67 (clone name: EPR3610, #ab92742, lot: GR 220263-2) for immunohistochemical staining was purchased from Abcam
Rabbit monoclonal antibodies recognizing ATP1A1 (clone name: EP1845Y, #ab76020, lot: GR 3237646-15) for immunoblotting was purchased from Abcam
Mouse monoclonal antibodies recognizing GAPDH (clone name: 6C5, #ab8245) for immunoblotting was purchased from Abcam
Mouse monoclonal antibodies recognizing GCP16 (clone name: NO-2, #sc-101278, lot: F1515) for immunoblotting was purchased from Santa Cruz Biotechnology
Mouse monoclonal antibodies recognizing GLUT3 (clone name: G-5, #sc-74399) for immunoblotting was purchased from Santa Cruz Biotechnology
Mouse monoclonal antibodies recognizing Flag tag (clone name: M2, #F3165) for immunoblotting was purchased from Sigma
Rabbit polyclonal antibodies recognizing GLUT1 pS226 (#ABN991) for immunoblotting was purchased from Sigma
Mouse monoclonal antibodies recognizing GST (clone name: 26H1, #2624, lot: 5) for immunoblotting was purchased from Cell Signaling Technology
Rabbit monoclonal antibodies recognizing hemagglutinin (HA) tag (clone name: C29F4, #3724, lot: 8) for immunoblotting was purchased from Cell Signaling Technology
Rabbit monoclonal antibodies recognizing Calnexin (clone name: C5C9, #2679, lot: 6) for immunoblotting was purchased from Cell Signaling Technology
Rabbit monoclonal antibodies recognizing cleaved PARP (clone name: D64E10, #5625, lot: 13) for immunohistochemical staining was purchased from Cell Signaling Technology
Rabbit monoclonal antibodies recognizing AKT pT308 (clone name: D25E6, #13038, lot: 7) for immunoblotting was purchased from

	Cell Signaling Technology<br>Rabbit monoclonal antibodies recognizing AKT (clone name: C67E7, #4691) for immunoblotting was purchased from Cell Signaling Technology<br>Rabbit polyclonal antibodies recognizing DHHC9 (#PA5-56868, lot: VE3001046) for immunoblotting/immunofluorescent staining was purchased from Thermo Fisher Scientific<br>Horseradish peroxidase-conjugated goat anti-mouse (#G-21040) secondary antibodies for immunoblotting was purchased from Thermo Fisher Scientific<br>Horseradish peroxidase-conjugated goat anti-rabbit (#G-21234) secondary antibodies for immunoblotting was purchased from Thermo Fisher Scientific<br>Alexa Fluor 488-conjugated goat anti-rabbit (#A-11008) secondary antibodies for immunofluorescent staining was purchased from Thermo Fisher Scientific<br>Alexa Fluor 594-conjugated goat anti-rabbit (#A-11012) secondary antibodies for immunofluorescent staining was purchased from Thermo Fisher Scientific
Validation	In general, we relied on data provided by the manufacturer's for validation as well as references in publications.<br>Rabbit monoclonal antibodies recognizing GLUT1 (#ab115730) for Flow Cyt (Intra), ICC/IF, WB, IHC-P in mouse, rat, human<br>Rabbit monoclonal antibodies recognizing tubulin (#ab176560) for Flow Cyt (Intra), WB, IHC-P, ICC/IF in human<br>Rabbit monoclonal antibodies recognizing Ki67 (clone name: EPR3610, #ab92742) for Flow Cyt (Intra), WB, IHC-P, ICC in human<br>Rabbit monoclonal antibodies recognizing ATP1A1 (#ab76020) for ICC/IF, Flow Cyt (Intra), WB, IHC-P in mouse, rat, human, Chinese hamster<br>Mouse monoclonal antibodies recognizing GAPDH (#ab8245) for WB, ICC/IF in mouse, rat, human<br>Mouse monoclonal antibodies recognizing GCP16 (#sc-101278) for WB, IP in mouse, rat, human<br>Mouse monoclonal antibodies recognizing GLUT3 (#sc-74399) for WB, IP, IF, ELISA in mouse, rat, human<br>Mouse monoclonal antibodies recognizing Flag tag (#F3165) for WB, IP<br>Rabbit polyclonal antibodies recognizing GLUT1 pS226 (#ABN991) for WB, IHC-P in mouse, human<br>Mouse monoclonal antibodies recognizing GST (#2624) for WB, IP, IF<br>Rabbit monoclonal antibodies recognizing hemagglutinin (HA) tag (#3724) for WB, IP, IHC-P, IF, Flow Cyt, ChIP<br>Rabbit monoclonal antibodies recognizing Calnexin (#2679) for WB, IHC-P, IF in in human, monkey<br>Rabbit monoclonal antibodies recognizing cleaved PARP (#5625) for WB, IP, IHC-P, IF, Flow Cyt in human, monkey<br>Rabbit monoclonal antibodies recognizing AKT pT308 (#13038) for WB, IP, IF, Flow Cyt in human, mouse, rat, monkey<br>Rabbit monoclonal antibodies recognizing AKT (#4691) for WB, IP, IHC-P, IF, Flow Cyt in human, mouse, rat, monkey, D. melanogaster<br>Rabbit polyclonal antibodies recognizing DHHC9 (#PA5-56868) for WB, IHC-P, ICC/IF in human

Eukaryotic cell lines

Policy information about cell lines

Cell line source(s)	Human GBM cell lines including U87, T98G, LN229, LN18, A172 were obtained from ATCC. Normal Human Astrocytes (NHAs) were obtained from Lonza.
Authentication	All cell lines used in this study were authenticated with STR profiling.
Mycoplasma contamination	All cell lines used in this study were negative for the tests of mycoplasma contamination.
Commonly misidentified lines (See ICLAC register)	No cell lines used in this study were found in the database of commonly misidentified cell lines maintained by ICLAC and NCBI Biosample.

Animals and other organisms

Policy information about studies involving animals; ARRIVE guidelines recommended for reporting animal research

Laboratory animals	The female 4-week-old Balb/c athymic nude mice were purchased from GemPharmatech (Nanjing, China). Mice were housed in a pathogen-free environment with the temperature maintained at 23 ± 2°C and relative humidity at 50 to 65% under a 12 h/12 h light/dark cycle with free access to food and water.
Wild animals	No wild animals were used.
Field-collected samples	No field-collected samples were used.
Ethics oversight	The animals were treated in accordance with the Guide for the Care and Use of Laboratory Animals published by the National Academy of Sciences and the National Institutes of Health. The use of animals in this study was approved by the Institutional Animal Care and Use Committee of the Center for Animal Experiments of the Institute of Biophysics, Chinese Academy of Sciences.

Note that full information on the approval of the study protocol must also be provided in the manuscript.

Human research participants

Policy information about studies involving human research participants

Population characteristics	Information about the patient sex, age, surgery date, overall survival time, and resection status are given in the source data of Supplementary Table 1. There were 37 males and 31 females, aged 32-80 years, with GBM. All patients had received standard clinical treatments.
Recruitment	Participants were recruited from the pool of GBM patients at the First Affiliated Hospital of Nanjing Medical University. The sample should be representative of the populations served by the hospital and reflect the general biological characteristics of GBM.
Ethics oversight	The use of patient specimens and the relevant database was approved by the Human Research Ethics Committee of the First Affiliated Hospital of Nanjing Medical University. The use of PDX cells derived from a GBM primary tissue was approved by the Human Research Ethics Committee of the Tianjin Medical University.

Note that full information on the approval of the study protocol must also be provided in the manuscript.
