# Supplementary material for: DHHC9-mediated GLUT1 S-palmitoylation promotes glioblastoma glycolysis and tumorigenesis
Source: Nat Commun. 2021 Oct 7;12:5872. doi: 10.1038/s41467-021-26180-4 (PMC8497546; doi:10.1038/s41467-021-26180-4)

Figure 1b

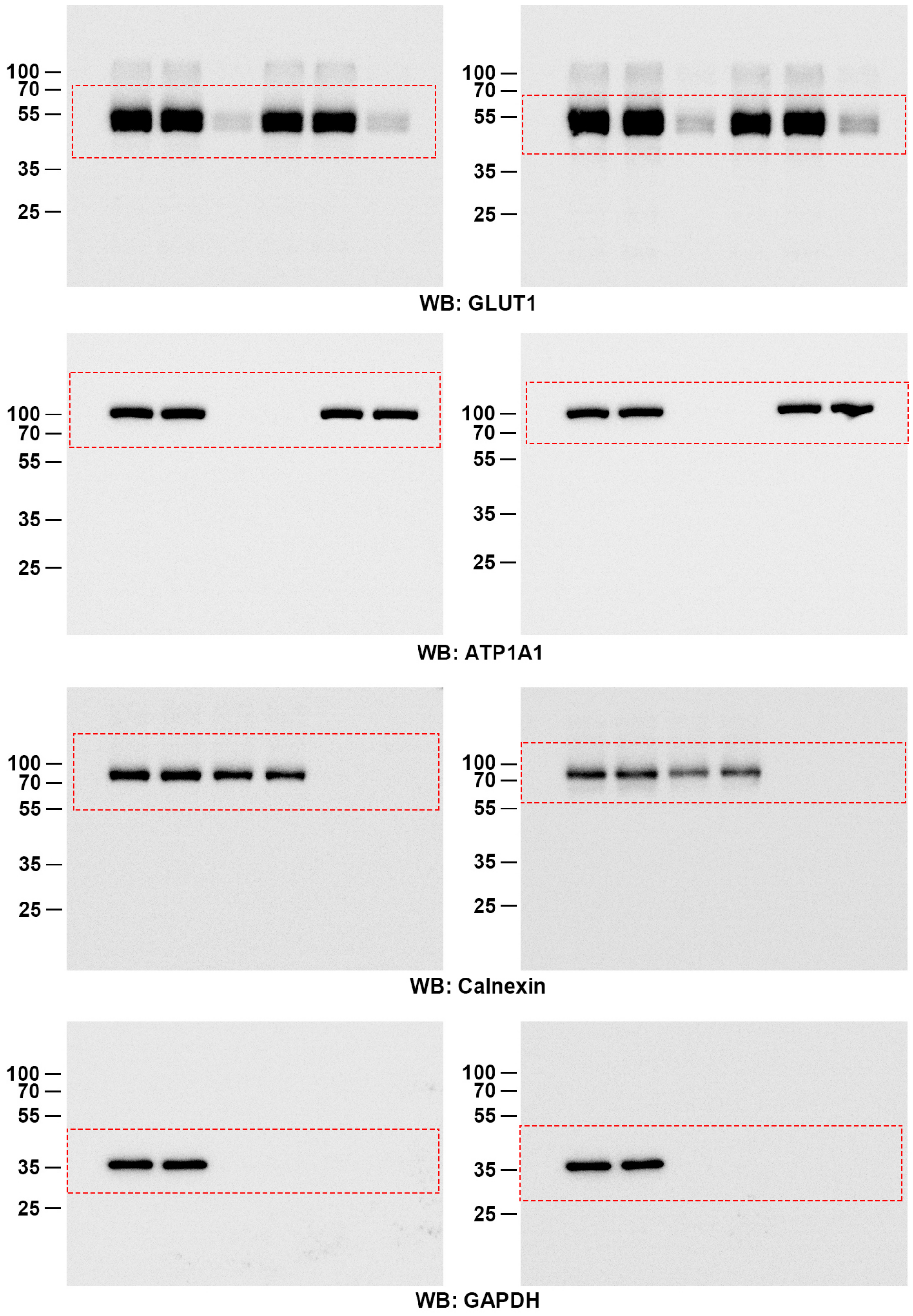

Figure 1c

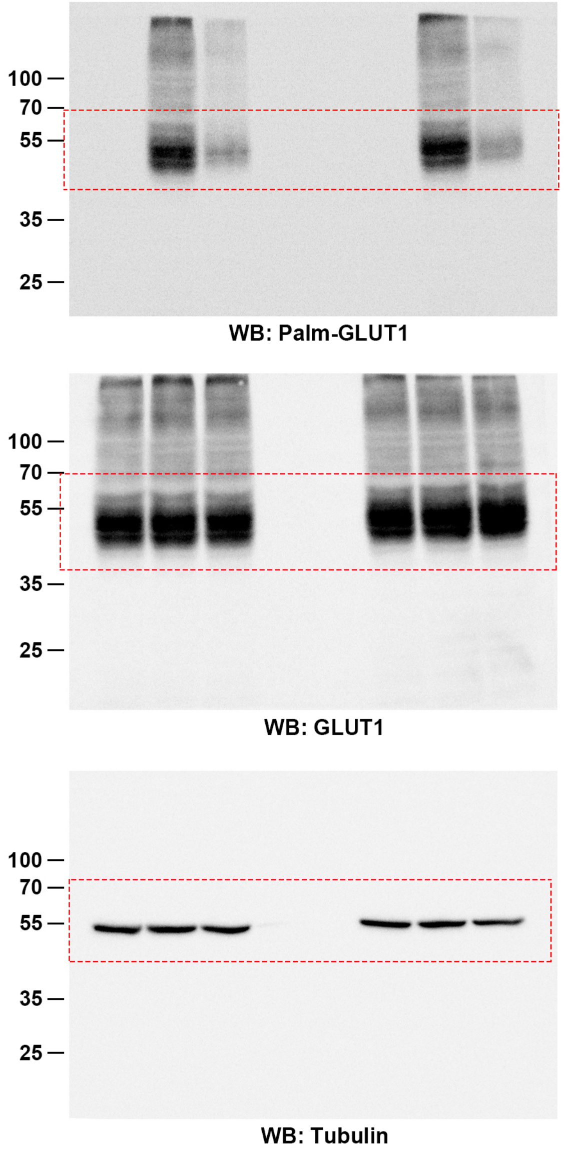

Figure 1d

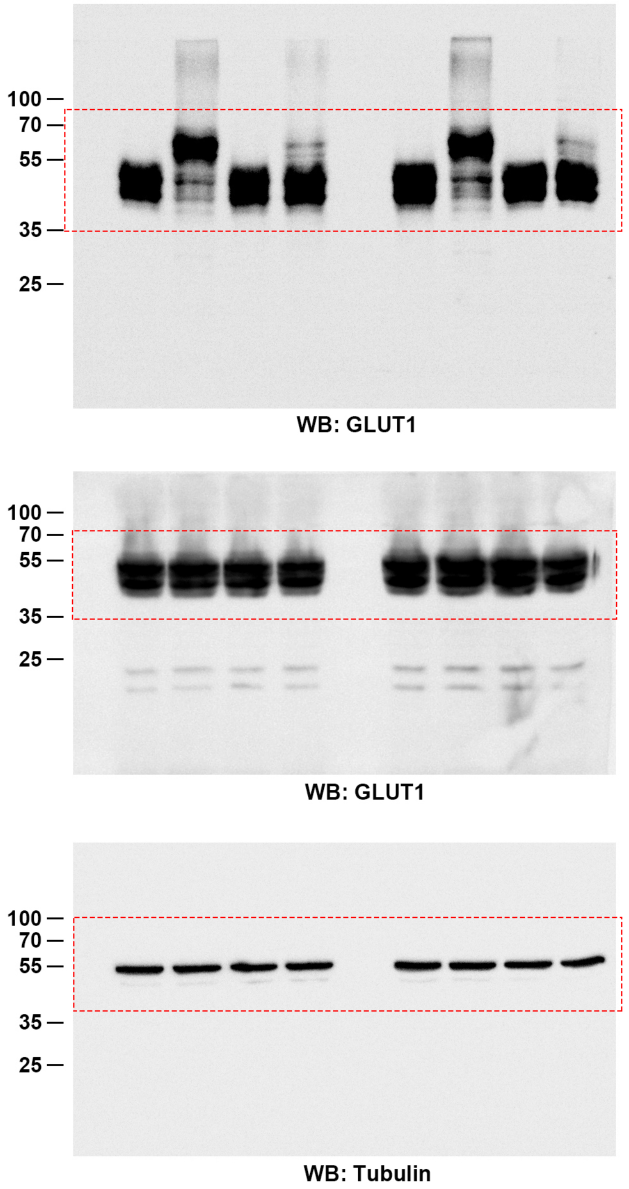

Figure 2a

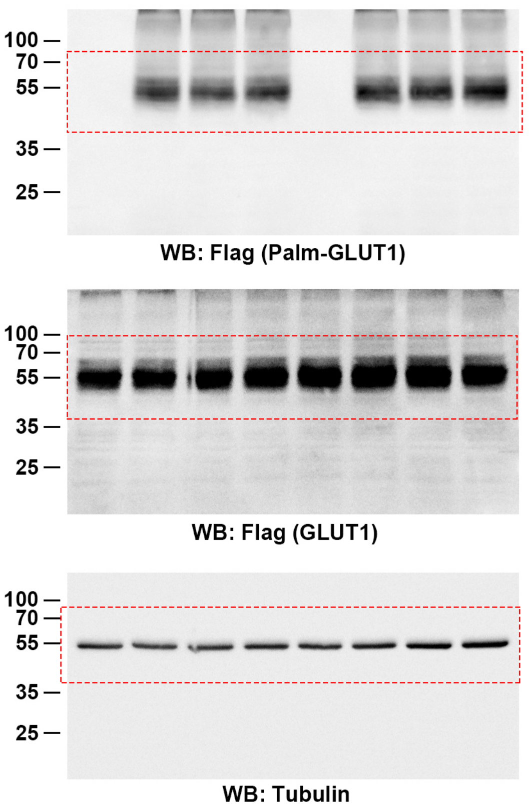

Figure 2b

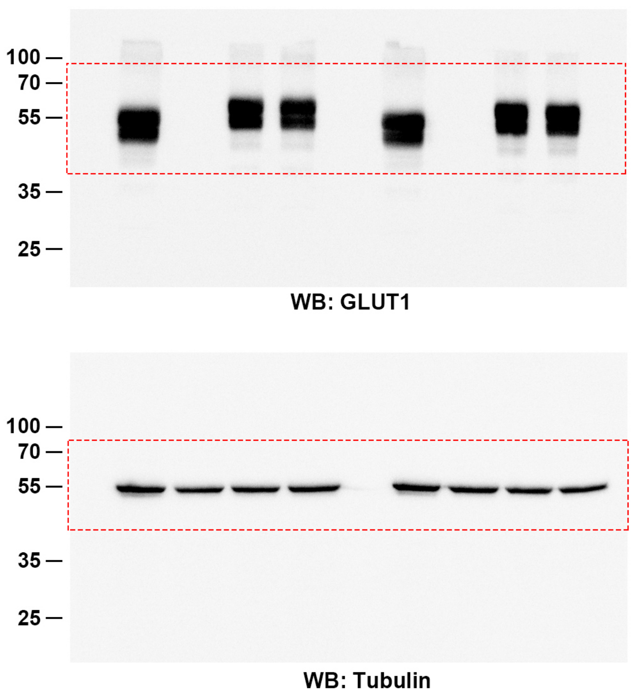

Figure 2c

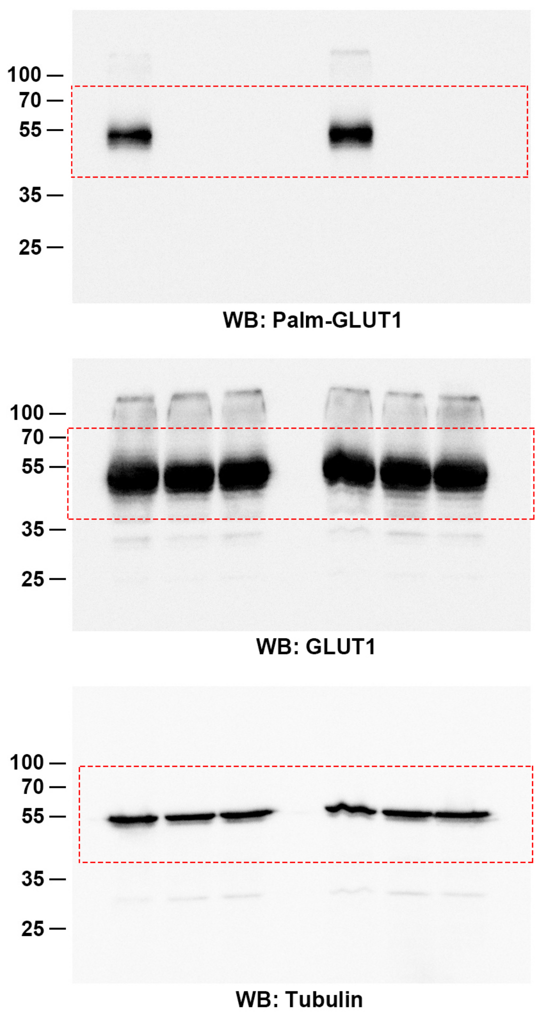

Figure 2d

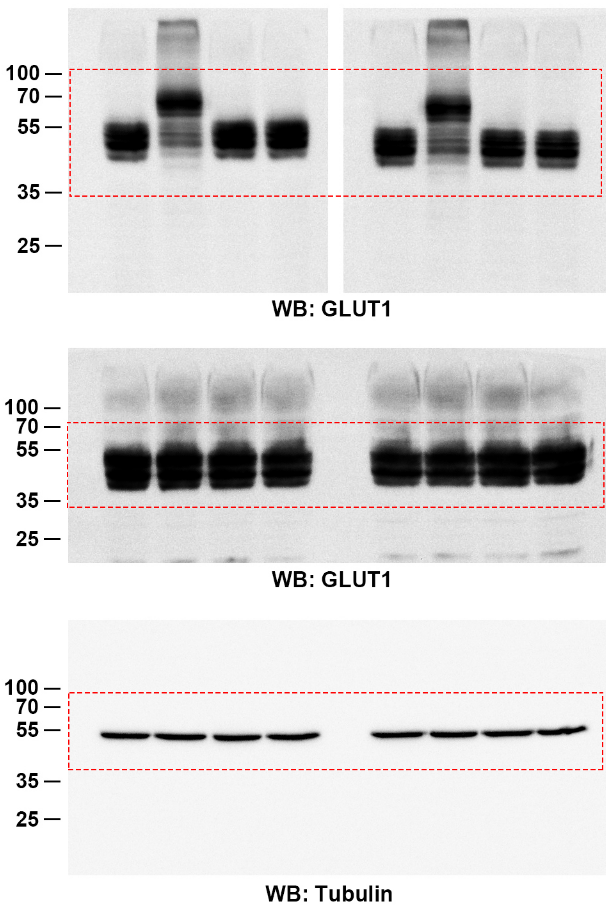

Figure 2f

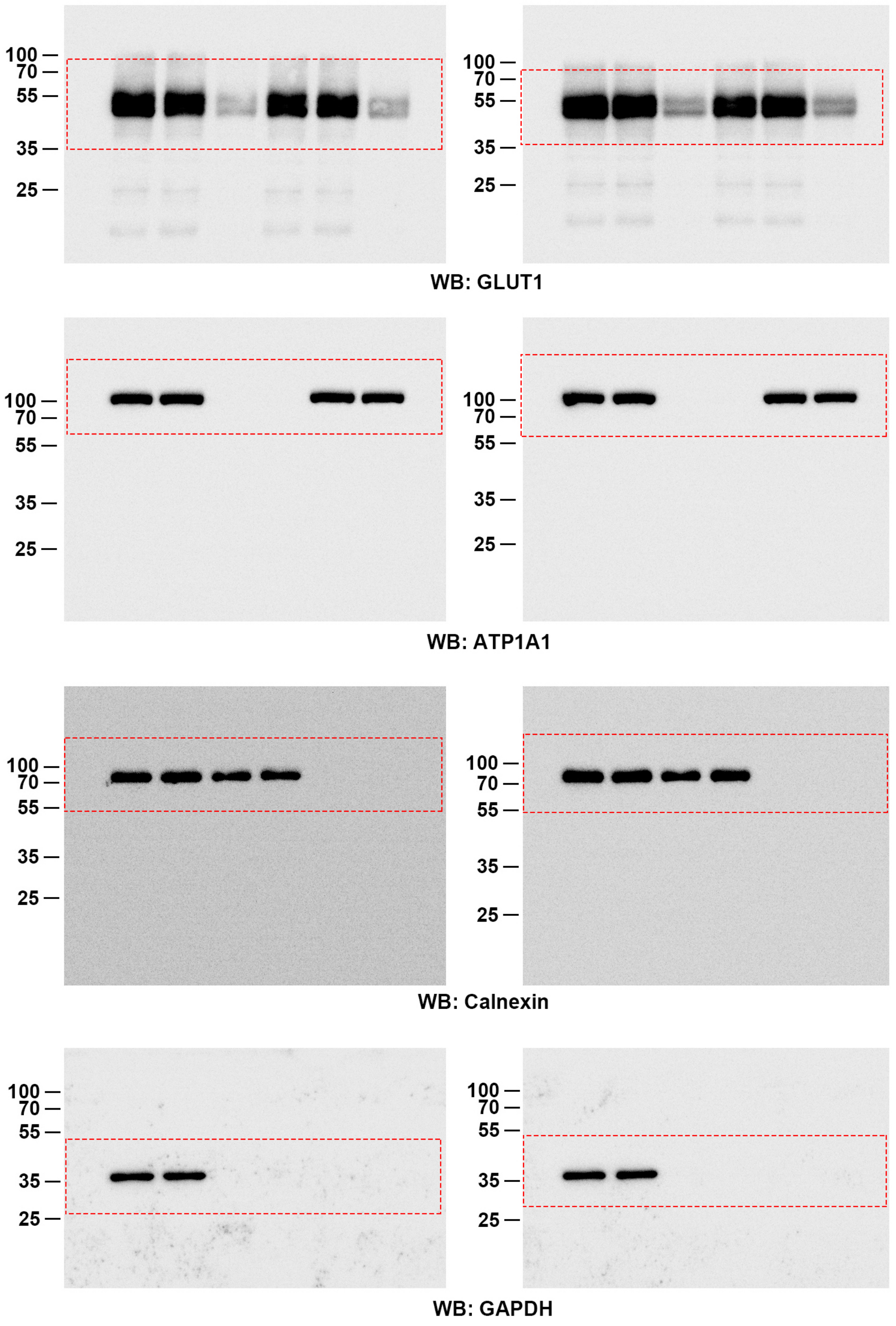

Figure 3a

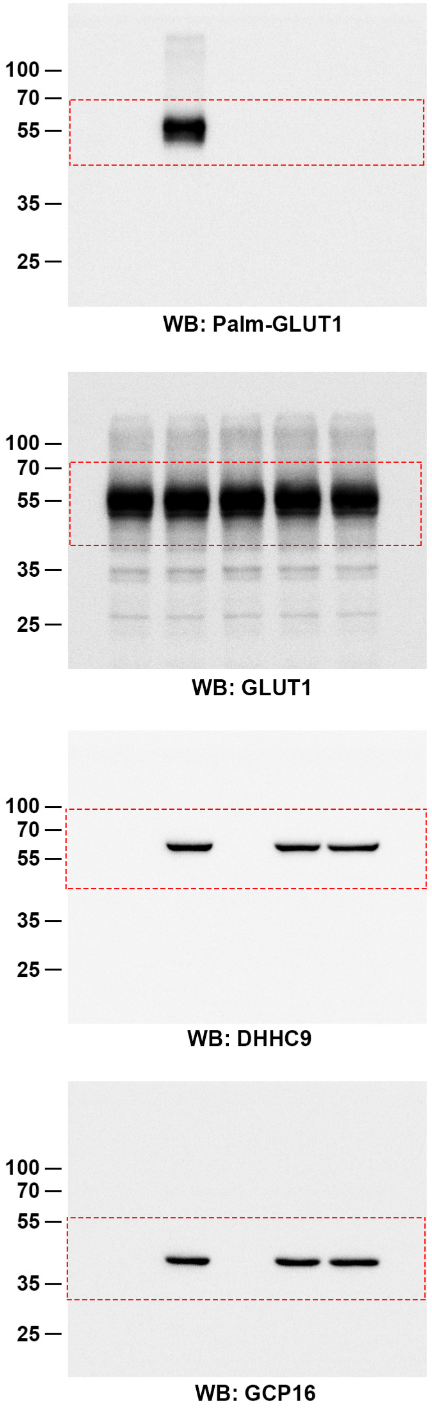

Figure 3b

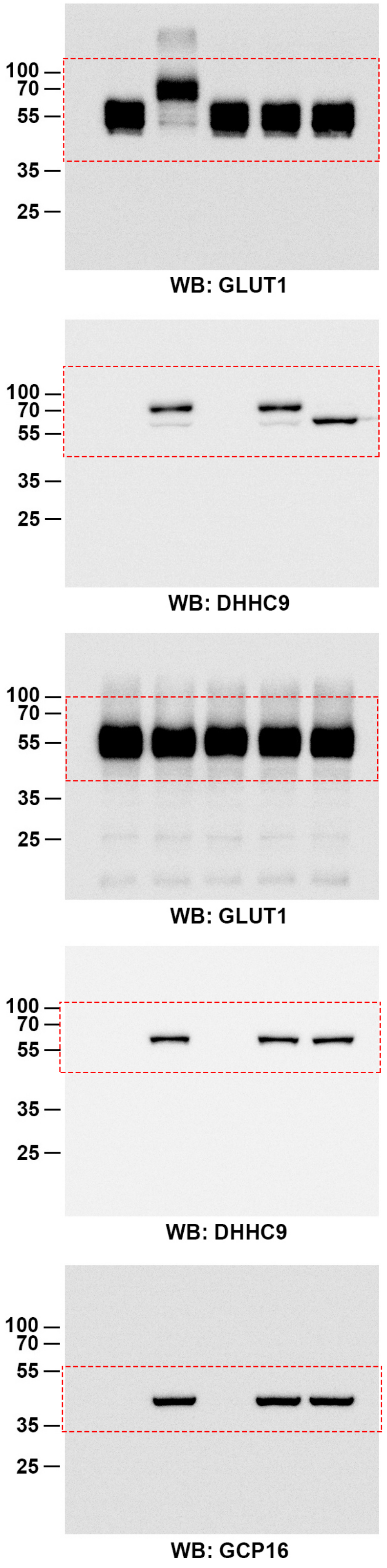

Figure 3c

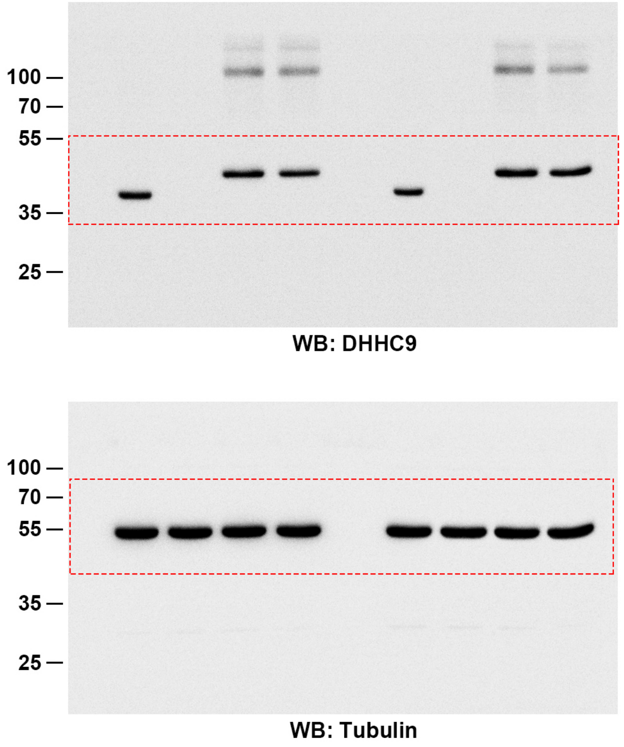

Figure 3d

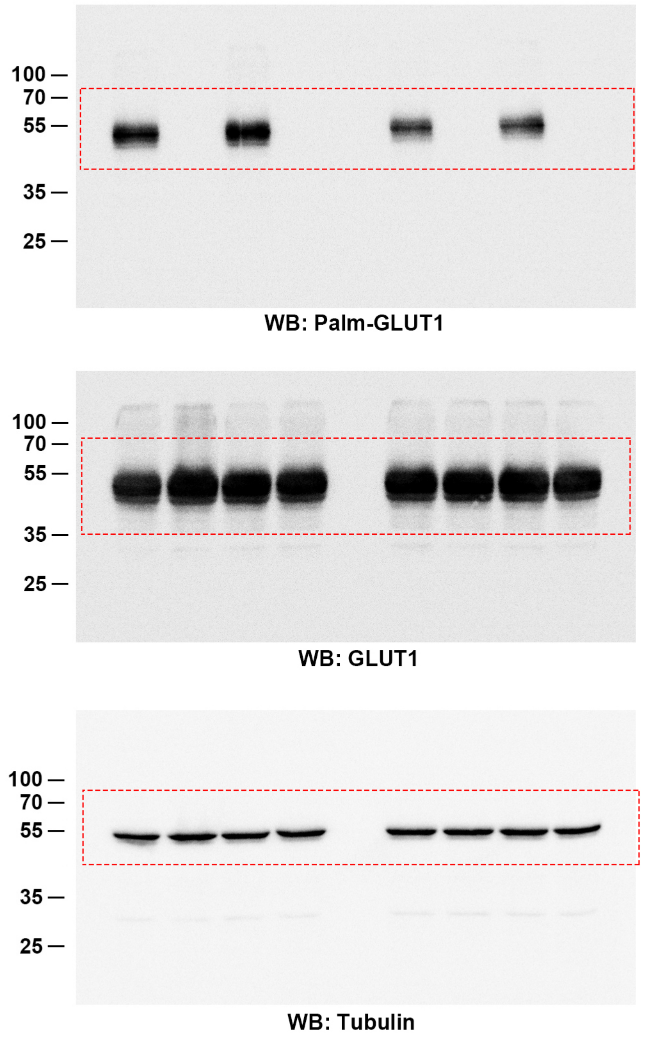

Figure 3g

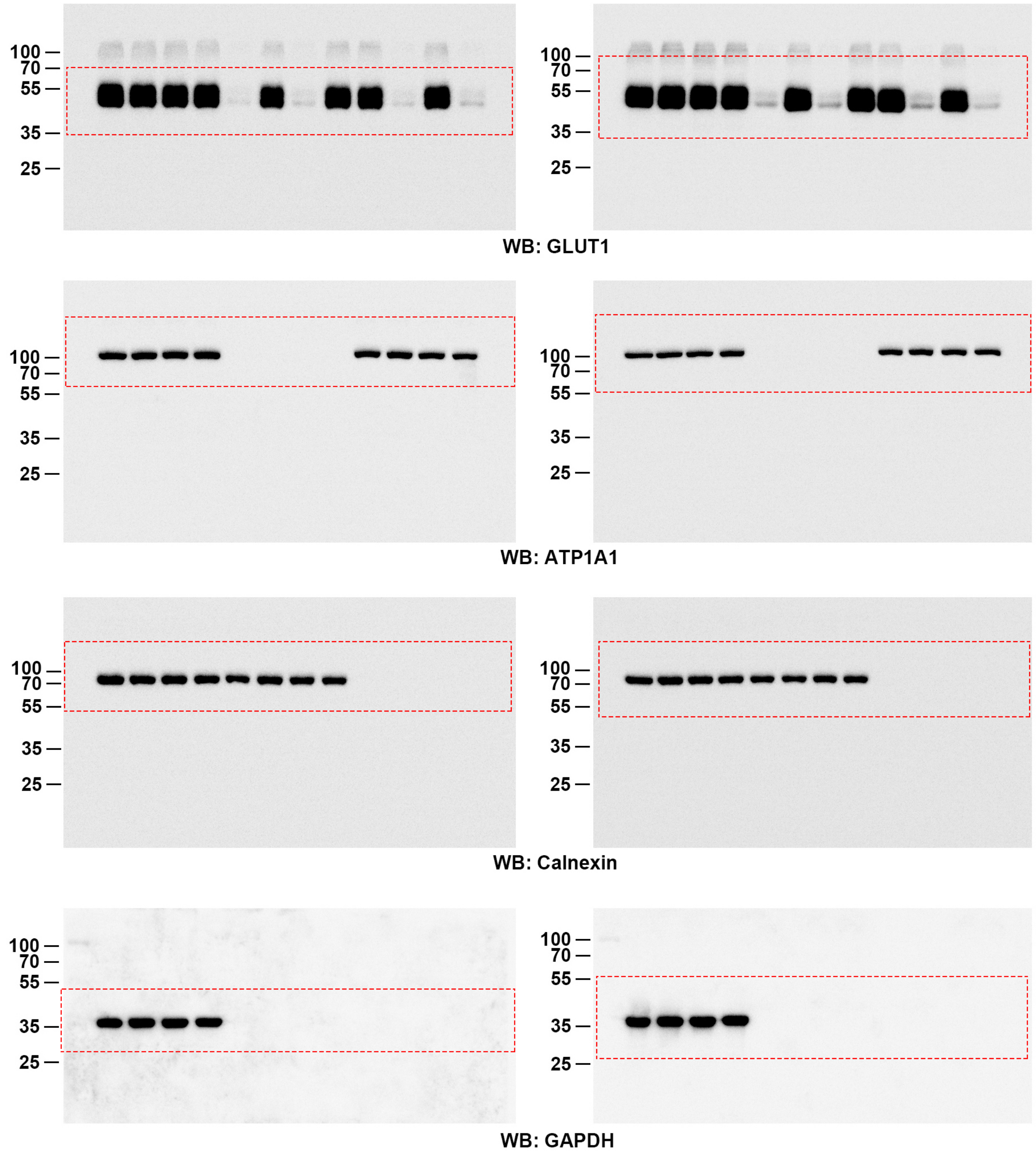

Figure 3e

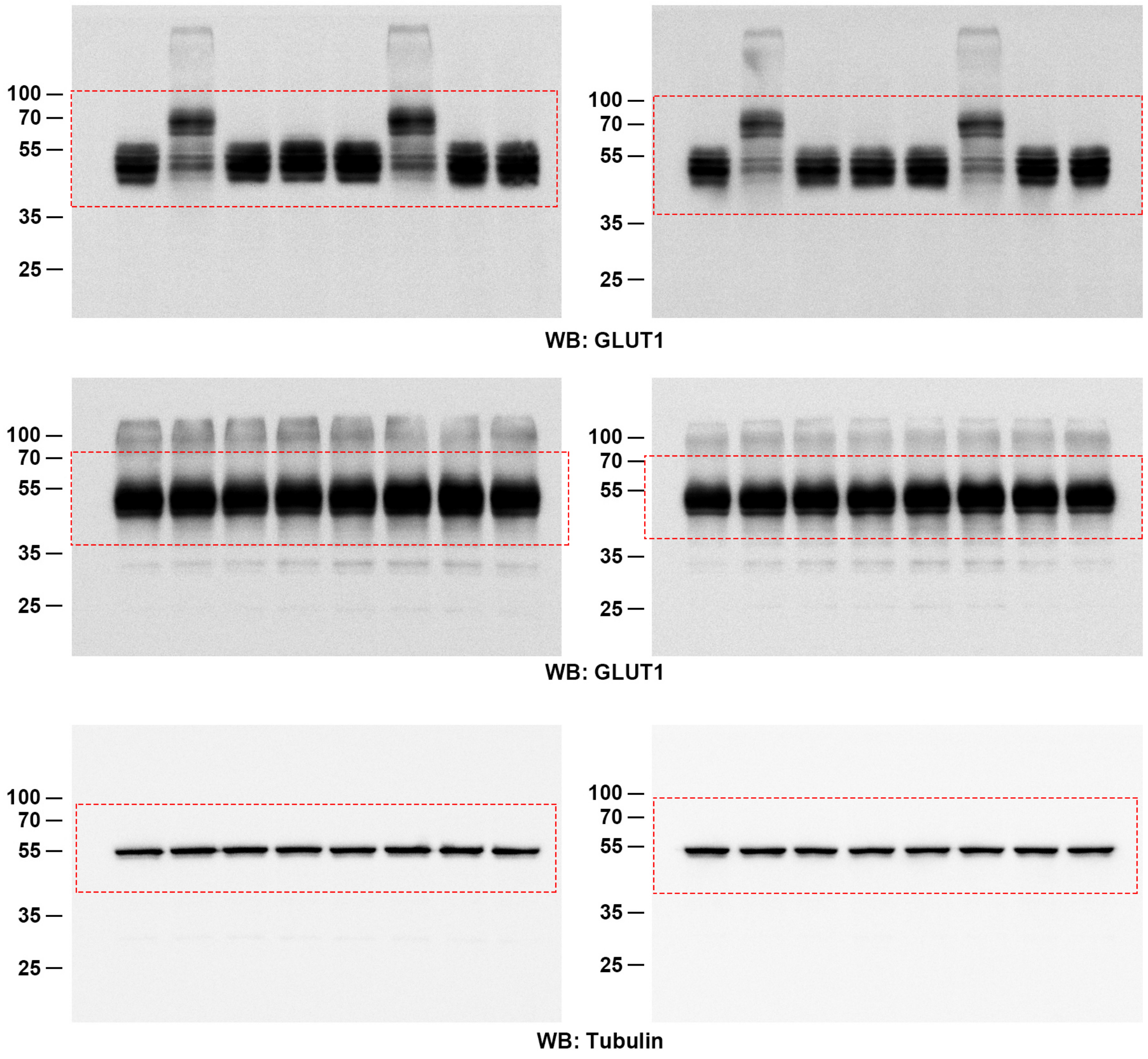

Supplementary Figure 1b

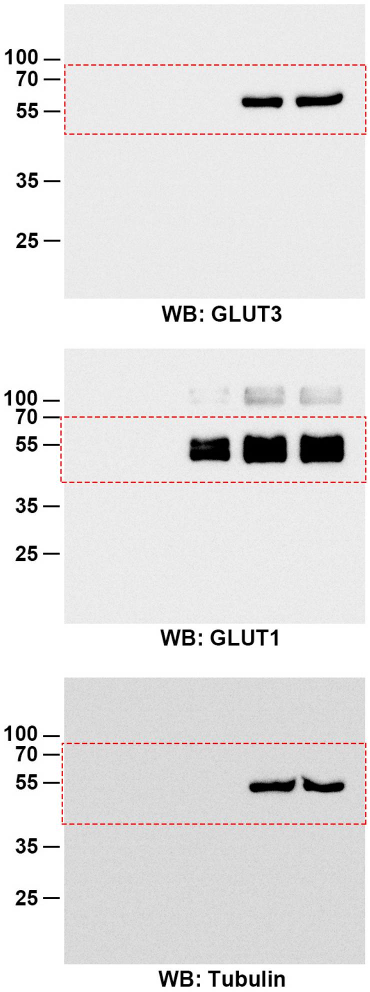

Supplementary Figure 1c

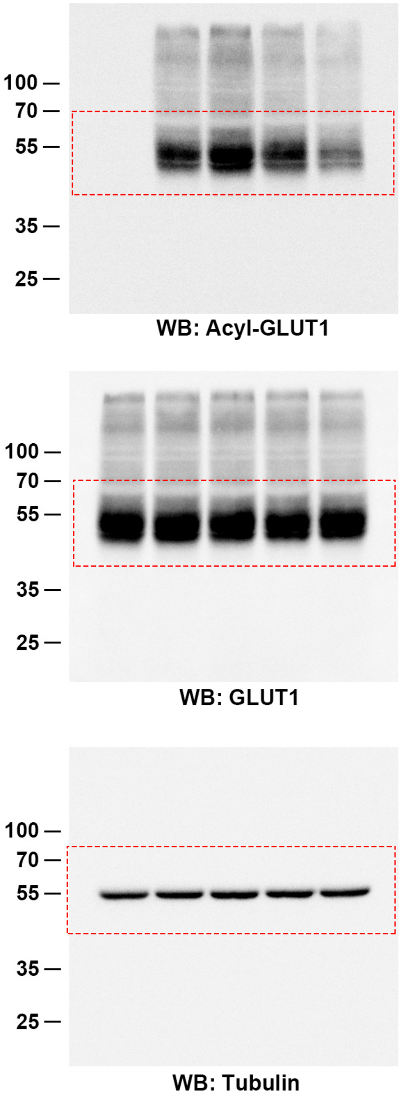

Supplementary Figure 1d

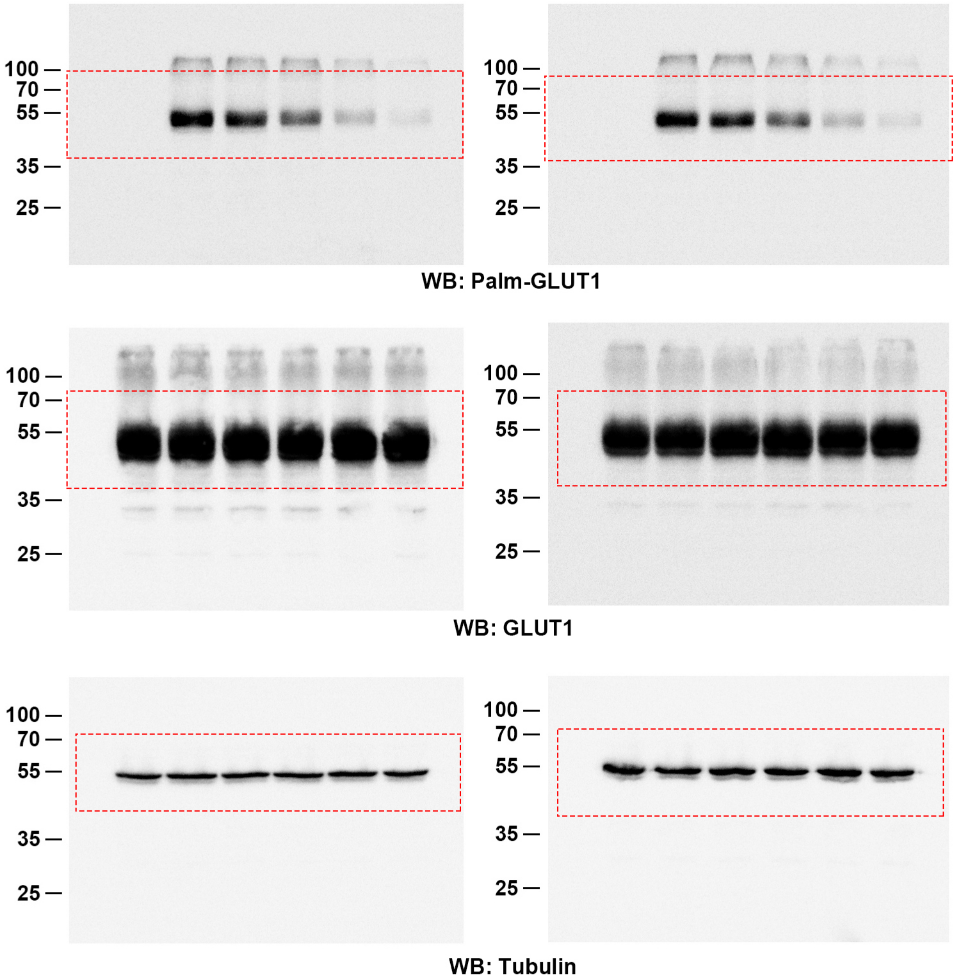

Supplementary Figure 3a

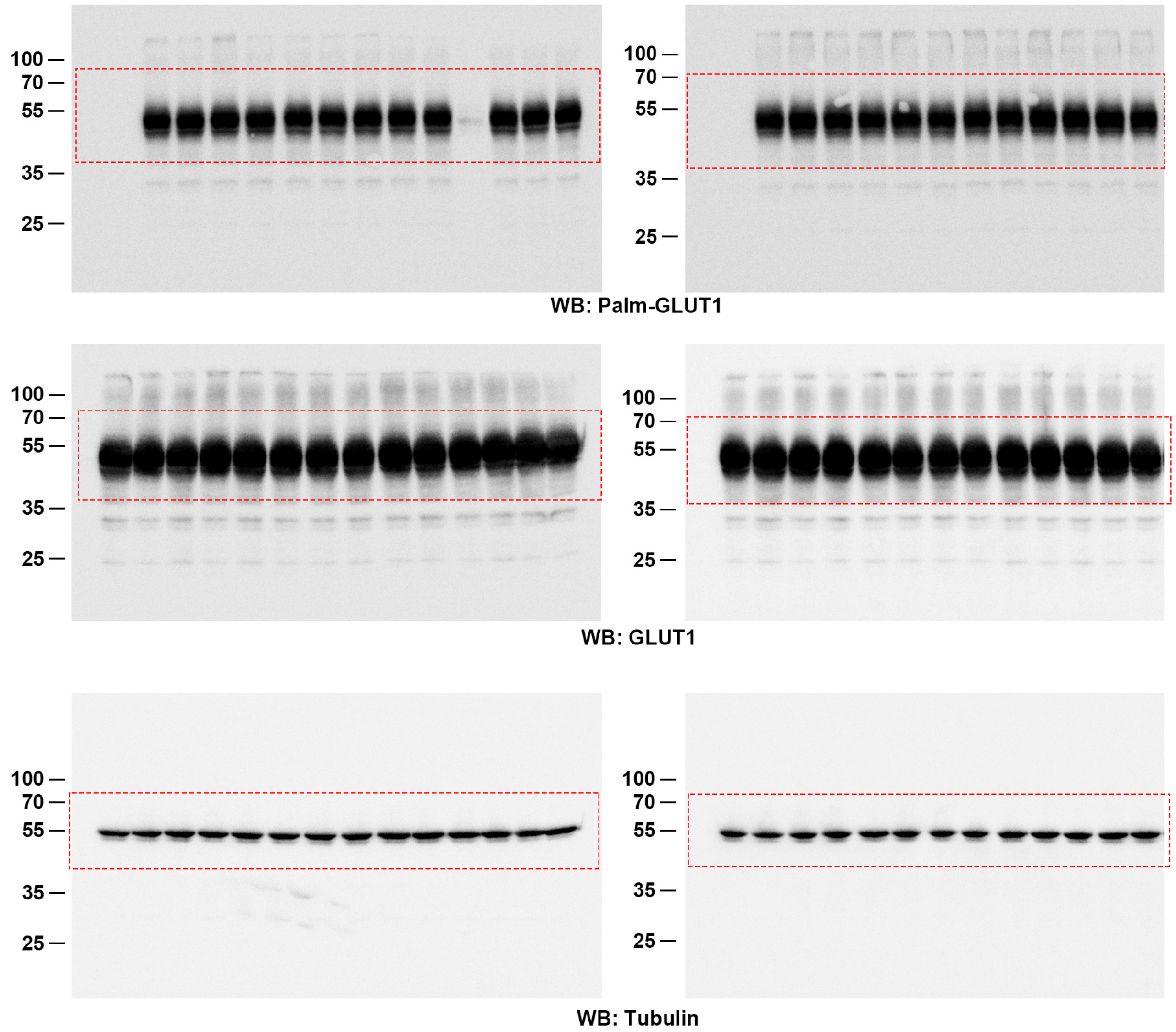

Supplementary Figure 3b

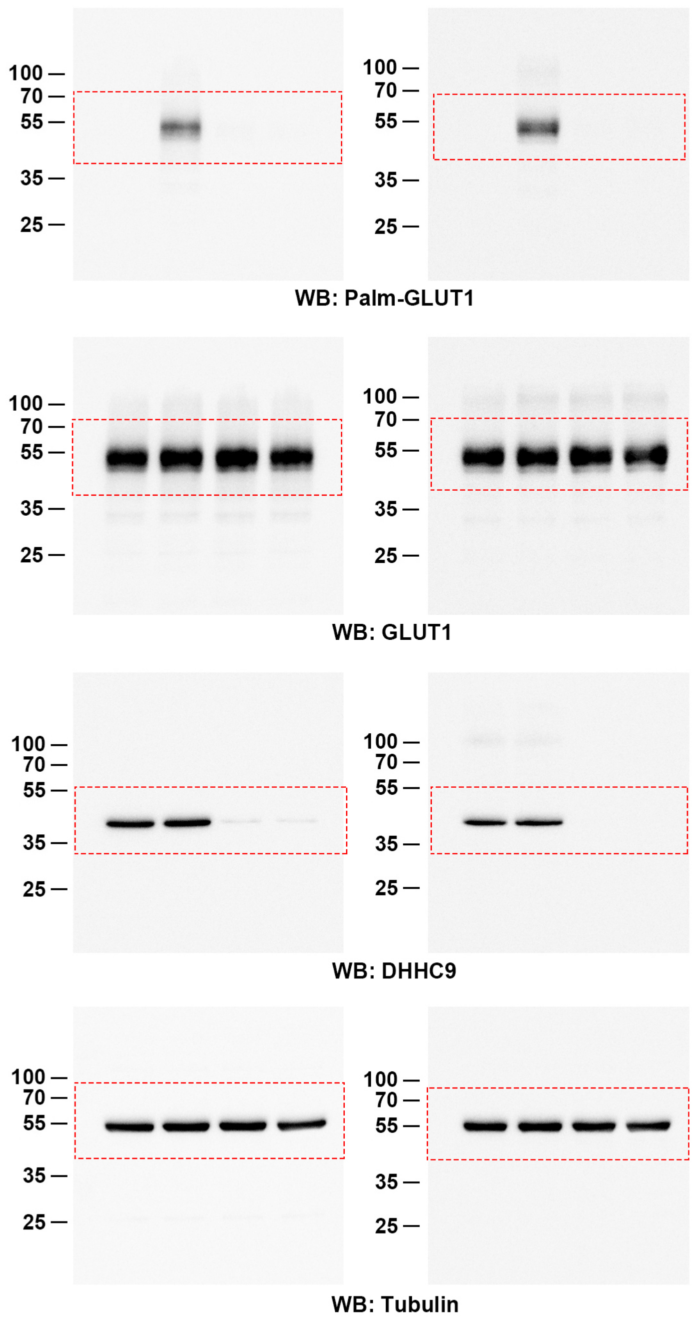

Supplementary Figure 3c

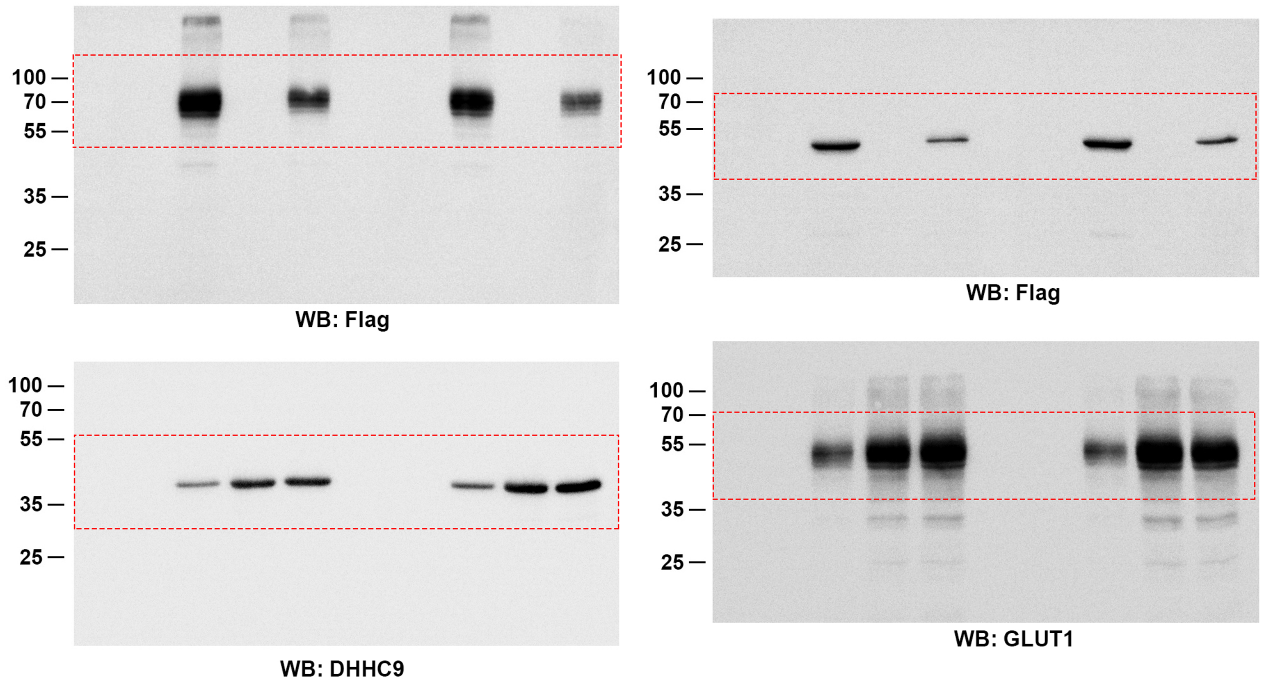

Supplementary Figure 3d

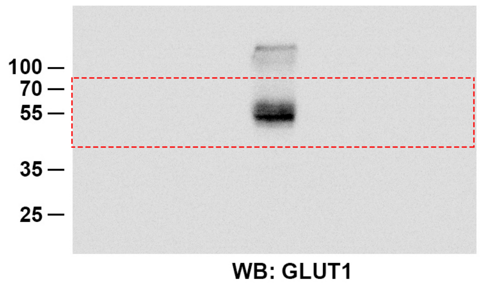

Supplementary Figure 3e

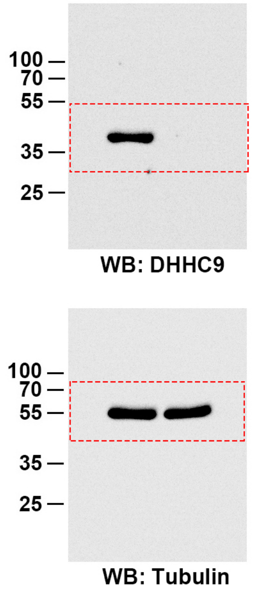

Supplementary Figure 4a

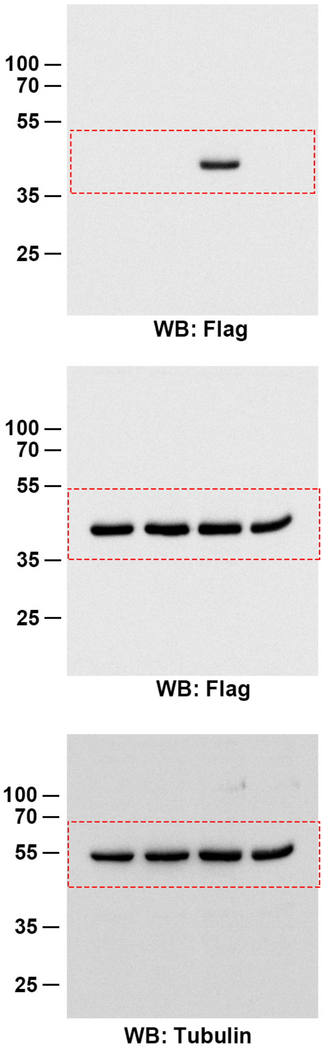

Supplementary Figure 4b

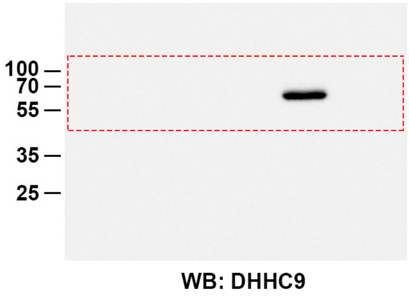

Supplementary Figure 4d

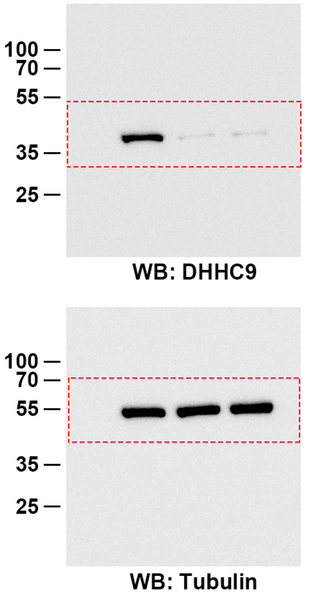

Supplementary Figure 4f

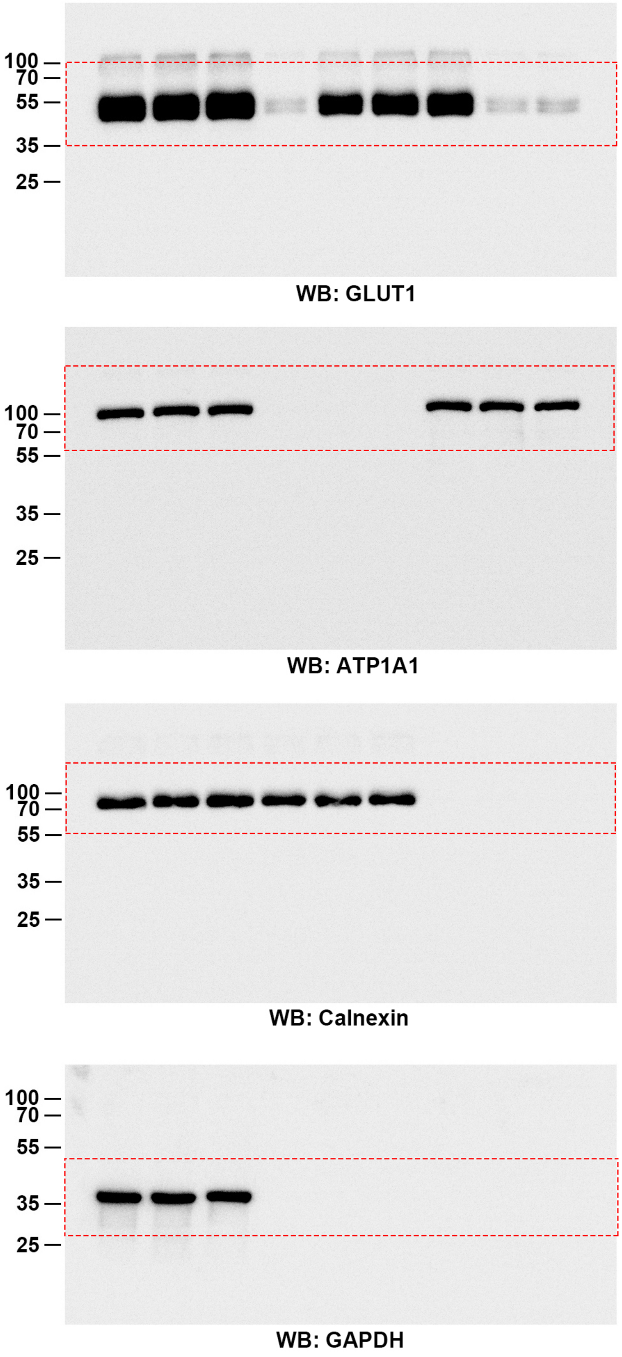

Supplementary Figure 5a

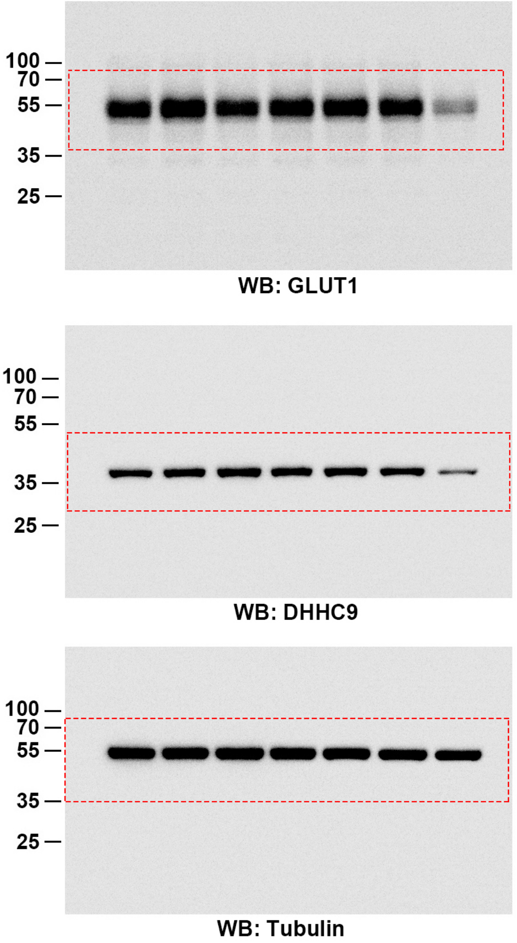

Supplementary Figure 5b

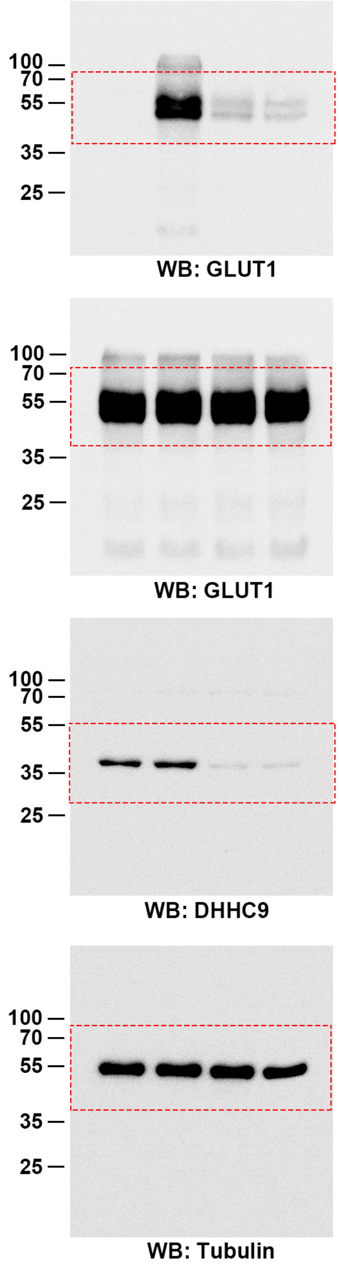

Supplementary Figure 5c

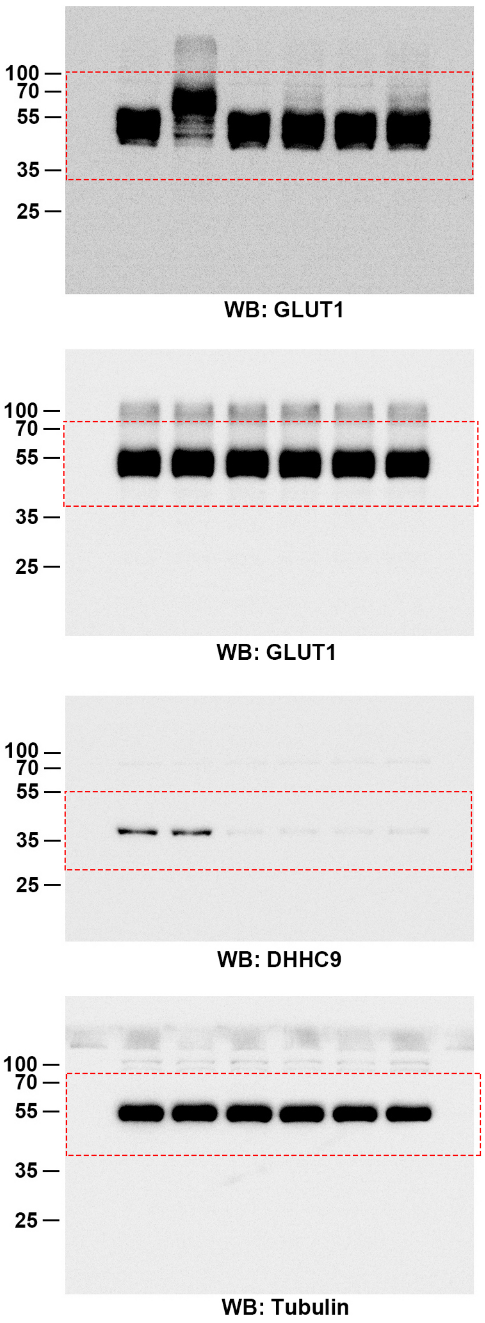

Supplementary Figure 5d

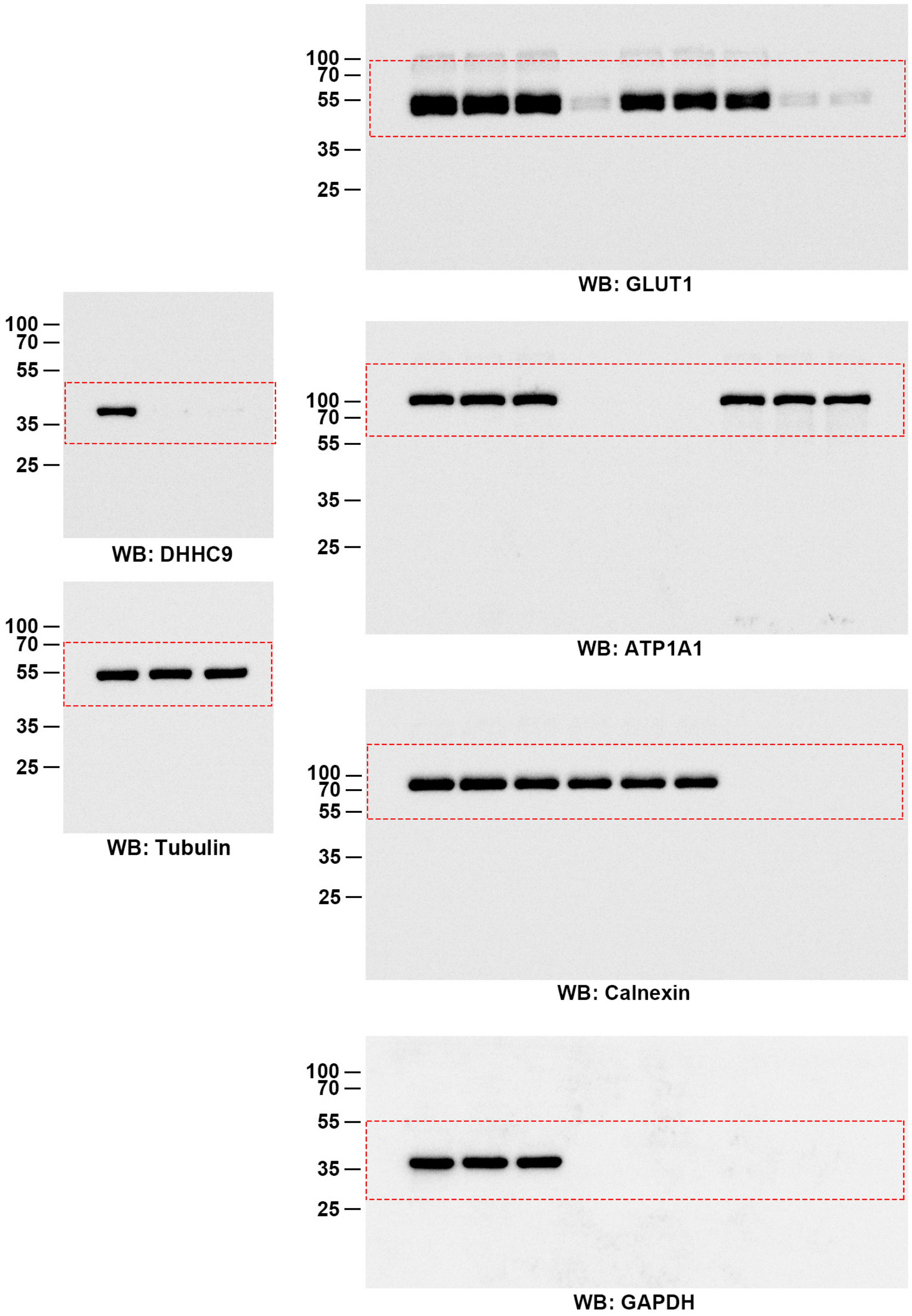

Supplementary Figure 5e

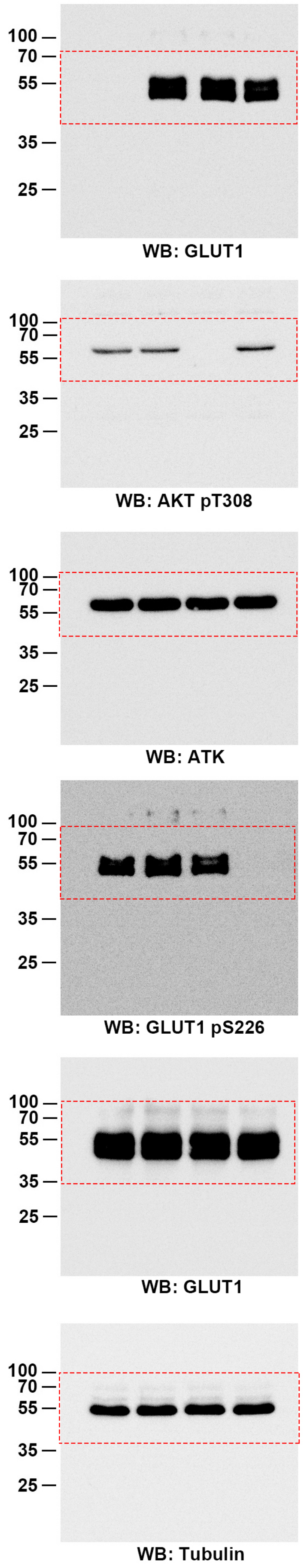

Supplementary Figure 6a

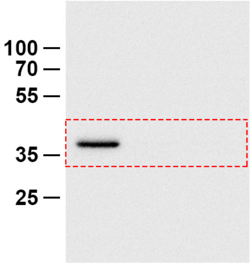

WB: DHHC9

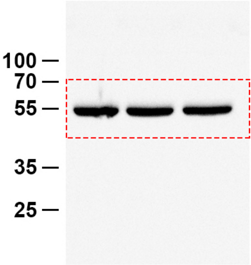

WB: Tubulin

Supplementary Figure 7a

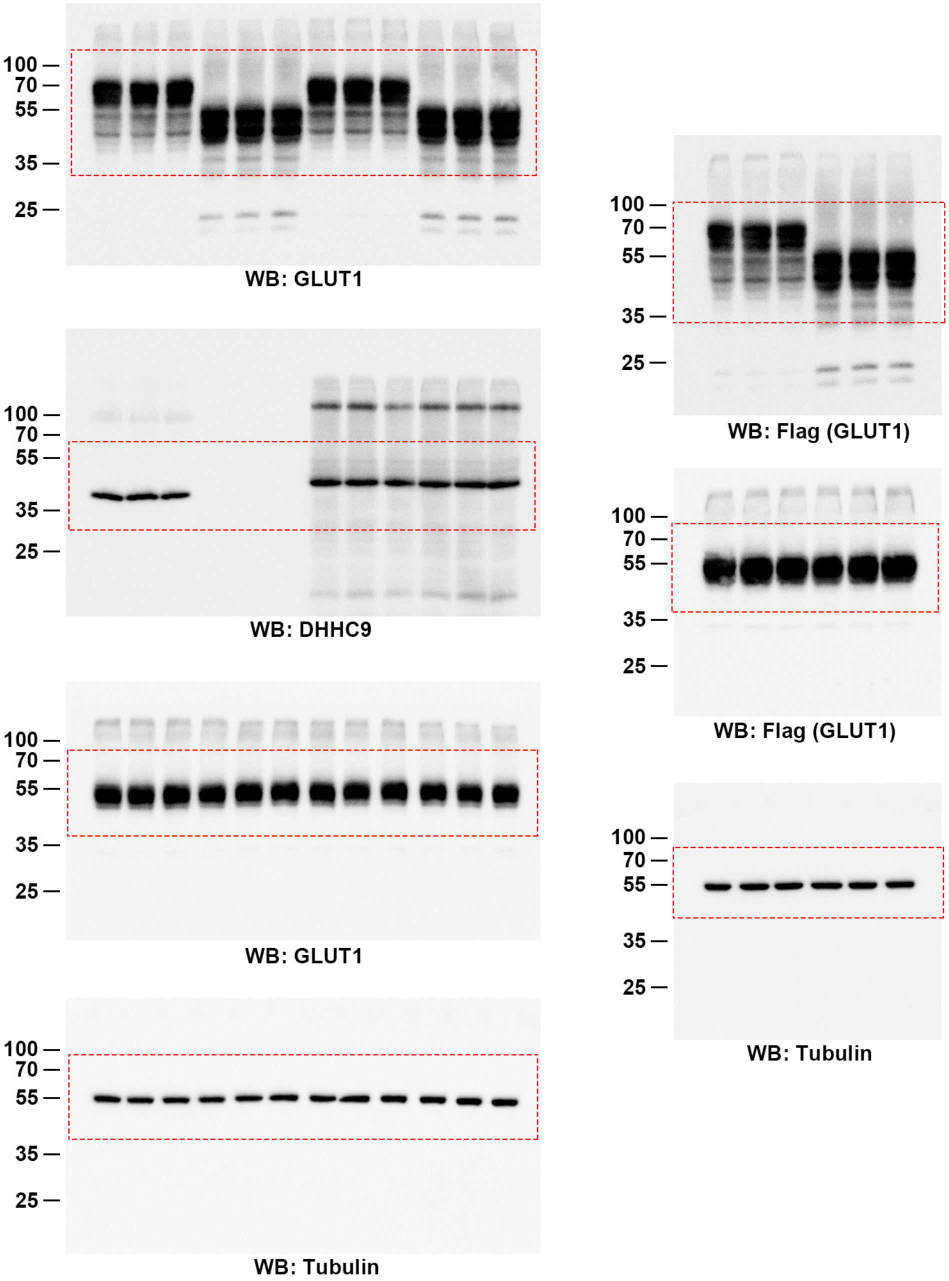

Supplement: Supplementary file 4 — Source Data [file 41467_2021_26180_MOESM4_ESM.zip › Uncropped immunoblotting.pdf]
